# Continuous neuromuscular blockade infusion for out-of-hospital cardiac arrest patients treated with targeted temperature management: A multicenter randomized controlled trial

**DOI:** 10.1371/journal.pone.0209327

**Published:** 2018-12-17

**Authors:** Byung Kook Lee, In Soo Cho, Joo Suk Oh, Wook Jin Choi, Jung Hee Wee, Chang Sun Kim, Won Young Kim, Chun Song Youn

**Affiliations:** 1 Department of Emergency Medicine, Chonnam National University Medical School, Gwangju, Korea; 2 Department of Emergency Medicine, Hanil General Hospital, Korea Electric Power Medical Corporation, Seoul, Korea; 3 Department of Emergency Medicine, The Catholic University of Korea School of Medicine, Seoul, Korea; 4 Department of Emergency Medicine, Ulsan University College of Medicine, Ulsan, Korea; 5 Department of Emergency Medicine, Hanyang University of Korea, Guri, Korea; 6 Department of Emergency Medicine, Asan Medical Center, University of Ulsan College of Medicine, Seoul, Korea; Universita degli Studi Magna Graecia di Catanzaro, ITALY

## Abstract

**Introduction:**

The aim of this trial was to investigate the effect of a continuous infusion of a neuromuscular blockade (NMB) in comatose out-of-hospital cardiac arrest (OHCA) subjects who underwent targeted temperature management (TTM).

**Methods:**

In this open-label, multicenter trial, subjects resuscitated from OHCA were randomly assigned to receive either NMB (38 subjects) or placebo (43 subjects) for 24 hours. Sedatives and analgesics were given according to the protocol of each hospital during TTM. The primary outcome was serum lactate levels at 24 hours after drug infusion. The secondary outcomes included in-hospital mortality, a poor neurological outcome at hospital discharge, changes in lactate levels, changes in the PaO_2_:FiO_2_ ratio over time and muscle weakness as assessed by the Medical Research Council (MRC) scale.

**Results:**

Eighty-one subjects (NMB group: median age, 65.5 years, 30 male patients; placebo group: median age, 61.0 years, 29 male patients) were enrolled in this trial. No difference in the serum lactate level at 24 hours was observed between the NMB (2.8 [1.2–4.0]) and placebo (3.6 [1.8–5.2]) groups (*p* = 0.238). In-hospital mortality and a poor neurologic outcome at discharge did not differ between the two groups. No significant difference in the PaO_2_:FiO_2_ ratio over time (*p* = 0.321) nor the MRC score (*p* = 0.474) was demonstrated.

**Conclusions:**

In OHCA subjects who underwent TTM, a continuous infusion of NMB did not reduce lactate levels and did not improve survival or neurological outcome at hospital discharge. Our results indicated a limited potential for the routine use of NMB during early TTM. However, this trial may be underpowered to detect clinical differences, and future research should be conducted.

## Introduction

Several trials have demonstrated that targeted temperature management (TTM) improves neurological outcomes in patients who remained comatose after ventricular fibrillation out-of-hospital cardiac arrest (OHCA). [1–4]. Shivering is commonly observed during TTM, which generates heat, increases metabolic rate, and consumes oxygen. Thus, shivering may attenuate the neuroprotective effect of TTM and prevent achievement of a target temperature [5, 6]. Therefore, shivering should be monitored and controlled to preserve the beneficial effects of TTM.

Neuromuscular blockade (NMB) stops shivering and is commonly employed [1,2]. Prolonged use also has the potential to cause critical illness polyneuromyopathy and mask clinical seizure or myoclonus during post-cardiac arrest care [7]. Neither the American Heart Association nor European Resuscitation Council recommended the routine use of NMB during TTM in their 2015 guidelines [3, 4]. A randomized controlled trial comparing the effectiveness between TTM at 33°C and 36°C used NMB to control shivering on an as-needed basis [8]. Recent clinical studies reported that a continuous infusion of NMB was associated with increased hospital survival and had beneficial effects on intensive care unit (ICU) survival in cardiac arrest patients treated with TH [9, 10]. Improved tissue perfusion and decreased metabolic demand were proposed as possible mechanisms involved in the decreased lactate levels after a continuous infusion of NMB [9]. However, these studies are only associative. A recent randomized clinical trial proved that continuous NMB reduces shivering and drug requirements and also shortens the time to awakening [11]. One multicenter registry study demonstrated that as-needed NMB was associated with a good neurological outcome [12].

We conducted a multicenter randomized controlled trial that investigated the effect of a continuous NMB infusion for 24 hours within 6 hours after the return of spontaneous circulation (ROSC) in comatose OHCA survivors treated with TTM.

## Methods

### Trial design

This clinical trial studied the effect of a continuous infusion of NMB versus an infusion with saline placebo in comatose OHCA survivors treated with TTM after ROSC. Subjects were recruited across eight emergency departments in the Republic of Korea and randomly assigned to one of two groups. The protocol was approved by the institutional review board of each participating hospital (The Catholic University of Korea, Chonnam University of Korea, Hanil general Hospital, Ulsan University of Korea, Hanyang University of Korea, Asan medical Center). This trial was approved to follow up with all participants until hospital discharge. The protocol was registered at *www.clinicaltrials.gov* (unique identifier: NCT02790164) 3 days after the first participant was enrolled. The reason for the delay in registration was poor communication between the researchers regarding trial registration. The authors confirmed that all ongoing and related trials for this drug are registered.

### Patients

Adult (over 19 years of age), comatose OHCA patients with ROSC irrespective of the initial rhythm and etiology of cardiac arrest were consecutively screened. Eligible subjects underwent TTM with a target temperature of either 33°C or 36°C and were enrolled within 6 hours of ROSC. The primary exclusion criteria included pre-existing dementia, brain injury, cerebral performance category [CPC] scale ≥ 3, traumatic cardiac arrest, or being a member of a protected population (pregnant women and prisoners). Written informed consent was obtained from a legal surrogate before treatment and from each subject who regained consciousness after treatment.

### Randomization and intervention

After being screened for eligibility, subjects were randomly assigned 1:1 to be treated with either a continuous NMB infusion or a placebo. Randomization was generated using a computer-assisted sequence with permuted blocks of varying sizes and sealed opaque envelopes. Healthcare providers caring for the patients were aware of the assignment due to the clinical effect of NMB. The physicians assessing neurological outcomes, statisticians, and study administrators were unaware of the assignments.

Subjects assigned to the NMB group received a bolus of 0.6 mg/kg rocuronium (concentration: 10 mg/ml) at the beginning of TTM, followed by an infusion of 0.3–0.6 mg/kg/hr. This infusion continued for a total 24 hours and was then discontinued. Subjects assigned to the placebo group received isotonic saline without NMB for 24 hours after assignment. The required isotonic saline volume was calculated assuming that rocuronium was given at a rate of 0.5 mg/kg/h. Therefore, 1.2*(body weight) in ml was given. However, the placebo group was allowed to receive bolus NMB in cases of intractable shivering or asynchrony with the mechanical ventilator for patient safety. The NMB group was also allowed to receive bolus NMB administration per clinician discretion. Advanced critical care measures such as oxygenation, ventilation, glucose control, and hemodynamic optimization, were provided according to the standard guidelines [3,4].

### Outcomes

The primary outcome was serum lactate level at 24 hours after initiation of the infusion, irrespective of group. Missing data of serum lactate levels at 24 hours in subjects who died within 24 hours were imputed with the mean value of the lactate levels at 24 hours in patients (both NMB and placebo) who subsequently died before hospital discharge. Secondary outcomes included poor neurological outcome at hospital discharge, defined as a CPC of 3–5 or a modified Rankin scale (mRS) of 4–6, or in-hospital mortality [13, 14]. Other secondary outcomes were ICU stay, hospital stay, and changes in the PaO_2_:FiO_2_ ratio over time (0, 12, 24, and 36 hours). The safety outcome was muscle weakness, which was assessed using the Medical Research Council (MRC) scale to test three muscle groups in each arm and leg at the time of ICU discharge. The score for each muscle group ranged from 0 (paralysis) to 5 (normal strength), with the overall score ranging from 0 to 60 [15].

The final follow-up concluded on January 27, 2017.

### Statistical analysis

We estimated the sample size using data from a previous prospective observational study of adult OHCA subjects. The mean 24-hour lactate levels were 1.6 ± 1.0 mmol/L for the NMB group and 4.3 ± 3.8 mmol/L for the placebo group [9]. Based on these results, we estimated a mean difference of 2.0 mmol/L with a standard deviation of 3.15 mmol/L. We estimated the need for 40 subjects in each group with a two-sided test at an alpha of 0.05 and power of 80%. The sample size calculation from secondary clinical outcomes (in-hospital mortality, neurologic outcome, and length of stay) was performed by simulating 1,000 samples. Using the method proposed by Finkelstein and Schoenfeld [16], every participant in the NMB group was compared to every participant in the placebo group and assigned a number from this comparison according to the hierarchical nature of the clinical outcome. Within each sample, the score for each participant was computed based on comparing each participant in the NMB group with a participant in the placebo group. These values were further compared by the Mann–Whitney procedure within each 1,000 samples, and their p-values were recorded. A proportion of tests with a p-value<0.05 equal 80% was achieved, with a sample size of 35 subjects in each study group. Considering both sample size calculations, we chose a sample size of 40 subjects per group.

Primary analyses were performed in the modified intention-to-treat (ITT) population, defined as all randomly assigned subjects except those who withdrew consent for the use of their data or those who did not fulfil the inclusion criteria. Post-hoc analysis, according to the presence of shock, was performed in the per-protocol population, which excluded subjects who had no serum lactate data at 24 hours due to early death. We did not find a difference in the baseline characteristics between the NMB and placebo groups. Therefore, we performed an analysis of covariance to ensure the effect of covariates due to the small sample size. Variables with p<0.8 were entered into the analysis of covariance as covariates.

Categorical variables are presented as frequencies and percentages. Comparisons of categorical variables were performed using χ^2^ or Fisher’s exact tests, as appropriate. Normality tests were performed for continuous variables, and continuous variables are presented as the means with the standard deviation or as median values with interquartile ranges, as appropriate. The Mann–Whitney *U* test or independent *t*-test was conducted for comparisons of continuous variables. A linear mixed model analysis was conducted to assess changes in the lactate levels and changes in the PaO_2_:FiO_2_ ratio over time. Post-hoc analyses of the comparisons between the groups at each time point were performed using a pair-wise Mann–Whitney *U* test and adjusted using Bonferroni correction. All tests were two-sided, and a p-value of 0.05 or less was considered to indicate statistical significance. Data were analyzed using the PASW/SPSS^TM^ software, version 18 (IBM, Inc., Chicago, IL, USA)

## Results

### Patients

**Fig 1:**
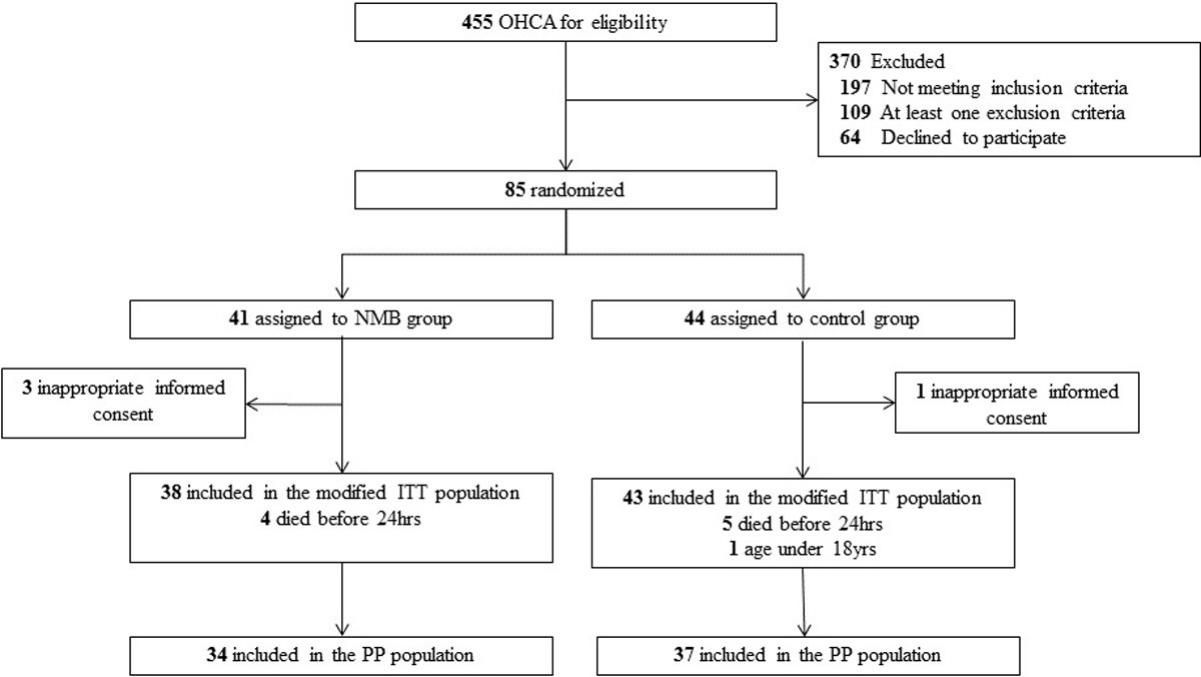
Screening, randomization, and analysis of study subjects.

Of the 455 OHCA patients screened between June 2016 and December 2016, 149 met the study inclusion criteria. A total of 85 subjects were enrolled in the study and randomly assigned to a group: 41 to the NMB group and 44 to the placebo group. The modified ITT population consisted of 38 subjects assigned to the NMB group and 43 patients to the placebo group “Fig 1”. The pre-randomization characteristics, cardiac arrest characteristics, and clinical characteristics on admission of the ITT population were similar (Table 1). The per-protocol population consisted of 34 patients assigned to the NMB group and 37 to the placebo group “Fig 1”. Two subjects in the NMB group and four subjects in the placebo group were treated at a target temperature of 36°C. Three subjects died within 12 hours after admission, six subjects died within 24 hours, and four subjects died within 36 hours. Four time points of the serum lactate levels were acquired in 68 (85%) subjects.

**Table 1:** Baseline characteristics, cardiac arrest characteristics, and clinical characteristics on ICU admission.

Variables	All patients (N = 81)	NMB (n = 38)	Placebo (n = 43)	p Value
Demographics				
Male sex, n (%)	59 (72.8)	29 (76.3)	30 (69.8)	0.508
Age, yr	62.0 (49.5–70.5)	65.5 (49.8–76.0)	61.0 (48.0–66.0)	0.074
Medical history				
Congestive heart failure, n (%)	2 (2.5)	1 (2.6)	1(2.3)	1.000
Ischemic heart disease, n (%)	5 (6.2)	4 (10.5)	1 (2.3)	0.181
Hypertension, n (%)	35 (43.2)	18 (47.4)	17 (39.5)	0.508
Diabetes, n (%)	23 (28.4)	12 (31.6)	11 (25.6)	0.550
Asthma or COPD, n (%)	8 (9.9)	6 (15.8)	2 (4.7)	0.138
Nephropathy, n (%)	9 (11.1)	3 (7.9)	6 (14.0)	0.490
Cerebrovascular attack, n (%)	7 (8.6)	4 (10.5)	3 (7.0)	0.701
Malignancy, n (%)	3 (3.7)	1 (2.6)	2 (4.7)	1.000
Previous myocardial infarction, n (%)	5 (6.2)	2 (5.3)	3 (7.0)	1.000
Smoking, n (%)	20 (24.7)	10 (26.3)	10 (23.3)	0.750
Cardiac arrest characteristics				
Witnessed, n (%)	58 (71.6)	29 (76.3)	28 (67.4)	0.619
Bystander CPR, n (%)	43 (53.1)	23 (60.5)	20 (46.5)	0.439
Primary shockable rhythm, n (%)	21 (25.9)	8 (21.1)	13 (30.2)	0.384
Time to ROSC, min, median (IQR)	26.0 (17.5–40.0)	26.0 (15.0–40.0)	33.0 (19.0–42.0)	0.443
Cardiac etiology, n (%)	46 (56.8)	22 (57.9)	24 (55.8)	0.690
Clinical characteristic on ICU admission				
Mean blood pressure, mmHg	86.8 ± 31.2	88.6 ± 30.6	85.2 ± 32.1	0.632
Glasgow Coma Scale, median (IQR)	3 (3–3)	3 (3–3)	3 (3–3)	0.594
SOFA	4 (4–12)	4 (4–10)	4 (4–13)	0.775
Shock, n (%)	51 (63.0)	23 (60.5)	28 (65.1)	0.669
ST elevation myocardial infarction, n (%)	9 (11.1)	4 (10.5)	5 (11.6)	1.000
Body temperature, °C	36.0 (35.9–36.3)	36.1 (36.0–36.3)	36.0 (35.9–36.4)	0.827
Serum lactate, mmol/L	7.7 (3.6–11.0)	7.8 (3.8–11.1)	7.3 (3.6–11.1)	0.854
Serum glucose, mg/dL	254 (196–315)	261 ± 110	273 ± 118	0.635
pH	7.10 ± 0.23	7.06 (6.92–7.29)	7.11 (6.92–7.28)	0.883
PaCO_2,_ mmHg	45.0 (34.8–66.9)	46.0 (35.0–68.6)	45.0 (34.0–65.5)	0.613
PaO_2,_ mmHg	98.0 (73.5–177.7)	114 (73–215)	91 (73–155)	0.192

NMB, neuromuscular blockade; COPD, chronic obstructive pulmonary disease; CPR, cardiopulmonary resuscitation; ROSC, restoration of spontaneous circulation; ICU, intensive care unit; SOFA, sequential organ failure assessment

### Intervention and drug administration

NMB was continuously infused in 38 subjects, and placebo infusion was initiated in 43 subjects. Four (10.5%) subjects receiving continuous NMB infusion and five (11.6%) subjects receiving a placebo died during the intervention period. An additional bolus of NMB was given to three subjects in the NMB group and 13 subjects in the placebo group (*p* = 0.012). The subjects who received the bolus of NMB in the placebo group required an average of three (IQR, 2–6) NMB infusions. The requirements of vasopressors, sedatives, and analgesics were not different between the NMB and placebo groups (Table 2).

**Table 2:** Medications during the first 24 hours of post cardiac arrest care.

Medications	All patients (N = 81)	NMB (n = 38)	Placebo (n = 43)	p Value
Vasopressors				
Dopamine, n (%)	39 (48.1)	19 (50.0)	20 (46.5)	0.754
Dose of dopamine, mg	769 (360–1,616)	646 (235–2,332)	883 (500–1,600)	0.844
Norepinephrine, n (%)	62 (76.5)	13 (81.6)	31 (72.1)	0.315
Dose of norepinephrine, mg	10.3 (0.7–30.0)	9.4 (1.2–34.1)	12.0 (0.6–27.0)	0.899
Vasopressin, n (%)	11 (13.6)	7 (18.4)	4 (9.3)	0.232
Dose of vasopressin, IU	40.0 (13.0–50.0)	27.3 (1.0–50.0)	49.4 (42.3–61.2)	0.186
Epinephrine, n (%)	4 (4.9)	2 (5.3)	2 (4.7)	1.000
Sedatives and analgesics				
Midazolam, n (%)	68 (84.0)	31 (81.6)	37 (86.0)	0.585
Dose of midazolam, mg	89.0 (52.4–154.3)	72.2 (52.8–180.0)	90.0 (50.7–145.9)	0.430
Propofol, n (%)	9 (11.1)	5 (13.2)	4 (9.3)	0.728
Dose of propofol, g	2.4 (1.8–5.0)	2.4 (1.7–6.2)	2.3 (1.8–3.7)	0.806
Remifentanil, n (%)	40 (49.4)	17 (44.7)	23 (53.5)	0.432
Dose of remifentanil, mg	9.6 (7.0–12.0)	9.6 (7.1–12.0)	9.6 (4.6–12.0)	0.619
Morphine, n (%)	2 (2.5)	1 (2.6)	1 (2.3)	1.000
Neuromuscular blockade				
Continuous rocuronium, mg	548 (318–720)	548 (318–720)	NA	
Bolus neuromuscular blockade	16 (19.8)	3 (7.9)	13 (30.2)	0.012
Bolus rocuronium, n (%)	5 (6.2)	0 (0.0)	5 (11.6)	0.057
Bolus vecuronium, n (%)	10 (12.3)	2 (5.3)	8 (18.6)	0.069
Bolus cis-atracurium, n (%)	2 (2.5)	1 (2.6)	2 (2.3)	1.000

NMB, neuromuscular blockade

### Changes in the serum lactate levels

**Fig 2:**
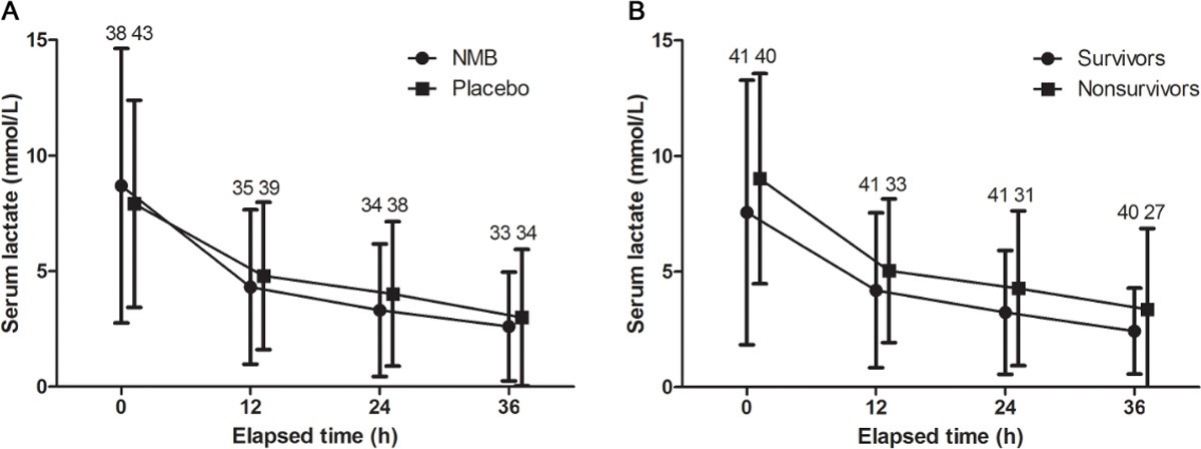
Changes in lactate levels over time, according to outcomes. Serum lactate levels significantly decreased over time. (A) Changes in lactate levels between the neuromuscular blockade (NMB) and placebo groups. Lactate levels were not different between the NMB and placebo groups (*p* = 0.565). (B) Changes in lactate levels in survivors and non-survivors. Survivors had a significantly lower serum lactate level (*p* = 0.047). However, post hoc analyses showed that there were no differences in the serum lactate levels at each time-point, between survivors and non-survivors. Numbers are included number of patients for analyses.

No difference in the serum lactate levels were observed at 24 hours between the NMB (2.8 [IQR: 1.2–4.0] mmol/L) and placebo (3.6 [IQR: 1.8–5.2] mmol/L) groups (*p* = 0.238). After adjustment for covariates, serum lactate at 24 hours did not differ by group (*p* = 0.255). Lactate levels significantly decreased over time in both groups. The changes in the lactate levels over time between the NMB and placebo groups were not different “Fig 2A; *p* = 0.148”. In-hospital mortality and neurologic outcomes were associated with the lactate levels “Fig 2B”.

No difference in the serum lactate levels was observed at 24 hours between the NMB (2.5 [1.1–4.1] mmol/L) and placebo (3.3 [1.8–5.8] mmol/L) groups in the per-protocol population (*p* = 0.216).

Post-hoc analysis according to the presence of shock showed that the serum lactate levels at 24 hours in subjects with shock were 2.3 (1.1–4.1) mmol/L in the NMB group and 4.1 (1.8–6.7) mmol/L in the placebo group (*p* = 0.068). In subjects without shock, the levels were 2.6 (1.3–5.1) mmol/L in the NMB group and 2.2 (1.3–4.0) mmol/L in the placebo group (*p* = 0.621).

### Clinical outcomes

The clinical outcomes are reported in Table 3. Overall, 40 subjects died in the hospital. Of those, 17 (44.7%) subjects were in the NMB group and 23 (53.5%) were in the placebo group (*p* = 0.432). No differences in ICU length of stay and hospital length of stay were found between the NMB and placebo groups.

**Table 3:** Primary outcome and clinical outcomes.

Outcomes	All patients (N = 81)	NMB (n = 38)	Placebo (n = 43)	p Value
Primary outcome				
Serum lactate at 24 hours	3.0 (1.7–4.3)	2.8 (1.2–4.0)	3.6 (1.8–5.2)	0.238
Clinical outcome				
In-hospital mortality	40 (49.4)	17 (44.7)	23 (53.5)	0.432
Poor neurologic outcome (CPC 3 to 5)	61 (75.3)	29 (76.3)	32 (74.4)	0.843
Poor neurologic outcome (mRS 4 to 6)	62 (76.5)	29 (76.3)	33 (76.7)	0.964
Length of stay				
ICU stay	7.4 (2.8–13.7)	8.0 (2.4–15.2)	6.4 (2.9–11.6)	0.481
Hospital stay	9.0 (3.0–18.0)	11.5 (2.0–21.3)	8.0 (3.0–18.0)	0.673
Discharge to home				
All patients	15 (18.5)	7 (18.4)	8 (18.6)	0.983
Hospital survivors	15/41 (36.6)	7/21 (33.3)	8/20 (40.0)	0.658

NMB, neuromuscular blockade; CPC, cerebral performance category; mRS, modified Rankin score; ICU, intensive care unit

**Fig 3:**
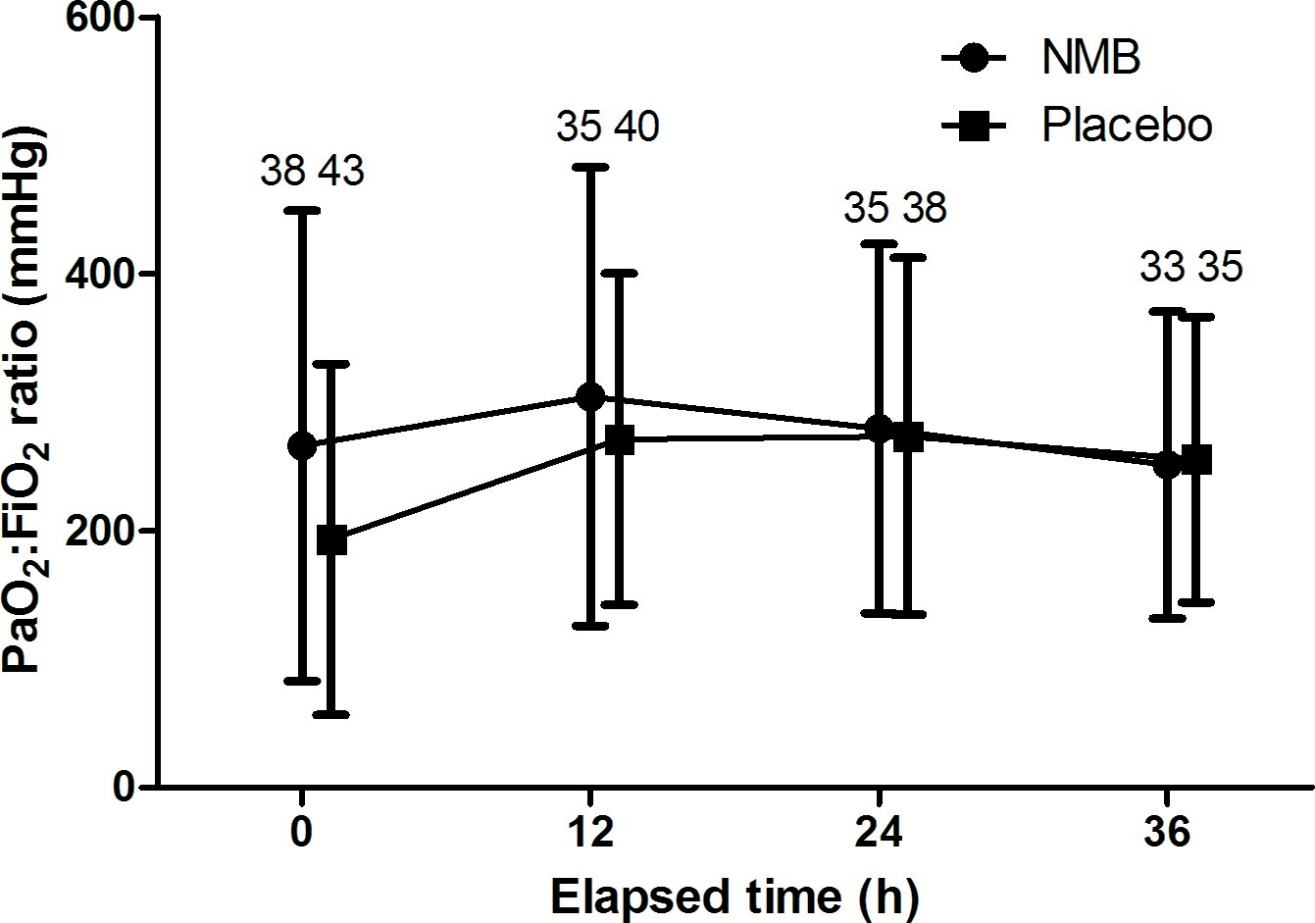
Changes in the PaO_2_:FiO_2_ ratio in the NMB and placebo groups. The PaO_2_:FiO_2_ ratio significantly changed overtime (*p* = 0.003). However, the PaO_2_:FiO_2_ ratios were not different between the neuromuscular blockade and placebo groups (*p* = 0.321).

The PaO2:FiO2 ratios in the entire cohort were 173 (IQR: 100–331), 281 (IQR: 162–397), 280 (IQR: 146–382), and 246 (IQR: 154–350) at 0, 12, 24, and 36 hours, respectively “Fig 3; p = 0.003”. The PaO_2_:FiO_2_ ratios were not different between the NMB and placebo groups (*p* = 0.321).

### Safety outcome

The MRC score was only measured in subjects with a CPC of 1–3 or an mRS of 0–4. Overall, 22 subjects had a CPC of 1–3 or an mRS of 0–4; of these, 11 subjects were in the NMB group and 11 were in the placebo group. The MRC score was not different between the NMB (60 [IQR: 48–60]) and placebo groups (60 [IQR: 60–60]) (*p* = 0.474).

## Discussion

In this multicenter randomized controlled trial, 24-hour lactate levels did not differ between subjects receiving a continuous infusion of rocuronium or placebo. This result was unchanged after adjusting with multiple covariates. Similarly, continuous infusion of rocuronium for 24 hours did not improve survival or neurological outcome at hospital discharge. The rate of intensive care unit-acquired weakness (ICU-AW) did not differ significantly between the two groups.

Neuromuscular blocking agents have potential advantages in treating critically ill subjects. These agents may improve chest wall compliance, eliminate ventilator dyssynchrony, reduce intra-abdominal pressure, prevent shivering, and prevent the elevation of intracranial pressure from airway stimulation [17, 18]. Disadvantages include potential masking of clinical seizures, interfering with clinical examinations after cardiac arrest (leading to the development of prolonged ICU-AW) and prolonging mechanical ventilation [19]. Hence, continuous infusion of NMB after CA remains controversial [4]. Two recently published observational studies reported that continuous infusion of an NMB could improve the clinical outcomes after OHCA treated with TH [9, 10] and a third demonstrated shortened the time to awakening and discharge from the ICU [11]. While a previous randomized study in subjects with ARDS found that continuous use of NMB for 48 hours resulted in better oxygenation compared to the placebo group, we did not demonstrate a difference in PaO2:FiO2 ratios in the groups [20].

Even though TTM after cardiac arrest is the standard treatment after OHCA, there is a great variability in the protocols used for sedatives and NMBs. In one systematic review, an NMB agent was routinely used to prevent shivering in 54 ICUs out of 68 IUCs and to treat shivering in eight ICUs [21]. In one ICU-based national survey from France, cisatraurium was used in 97% of subjects who were given NMB during TH [22].

Experimental and clinical studies confirm tissue hypoxia, characterized as a supply-dependent oxygen consumption, to be the cause of lactate elevation [23, 24]. Correcting this mismatch will decrease serum lactate levels and has been associated with survival and neurologic outcomes [25–27]. Salciccioli et al. suggested that continuous infusion of a NMB was associated with survival (adjusted OR for 7.23) and the 24-hour lactate levels (4.3 ± 3.8 mmol/L for no-NMB group versus 1.6 ± 1.0 mmol/L for NMB group) after CA [9]. We did not find a more robust reduction in the lactate levels in subjects treated with continuous NMB compared to those given a placebo.

A possible explanation for the difference between the two studies might be the baseline characteristics of the continuous NMB group. Subjects who received a continuous NMB infusion, as reported by Salciccioli et al., had shorter downtimes (13 minutes versus 26 minutes), more shockable primary rhythms (61% versus 21%), and lower levels of lactate on admission (4.9 mmol/L versus 7.8 mmol/L) than those of the participants of the present trial. Subject included in this trial may have had more severe ischemic reperfusion injury, which could also be the reason for the difference in the survival rates between the two studies (78% versus 56%). However, subjects enrolled in this trial at a relatively high rate (57% of all patients). Thus, our results may be more representative of all OHCA patients in Korea. The baseline characteristics and survival rates in this trial are similar to those of previous published data from the Korean Hypothermia Network registry [28].

One major strength of this trial is that life-sustaining treatment was not withdrawn, which is a common cause of death after OHCA [29]. Moreover, we could not find any significant differences in ICU-AW between the two groups.

There are several limitations to this trial, including a small sample size, which limited the power to detect differences in survival and poor neurological function. The primary outcome of this trial, 24-hour lactate levels, did not differ between groups. Lactate levels tended to be low in the NMB group, making it difficult to demonstrate a difference between groups. This trend was maintained in the shock group, and the lactate level difference between the placebo group and NMB group was 1.8 mmol/L. Moreover, there was an almost 10% reduction in in-hospital mortality (53.5% in the placebo group vs 44.7% in the NMB group). Given these concerns, increasing the sample size would be the only way to verify this result. One ongoing multicenter trial of continuous NMB after cardiac arrest in USA (ClinicalTrials.gov number, NCT02260258) with a combined analysis is planned and may offer additional information. Second, several types of NMB were administered as a bolus injection in the placebo group to treat shivering. Although these subjects did not received continuous infusion of NMB, it could be a confounding factor in this trial. Third, this trial was not blinded. Due to the inherent characteristics of NMB efficacy and patient safety, this was an open-label trial. Therefore, we cannot completely exclude the possibility the of effect of the physician’s discretion on the clinical practice. Fourth, stratifying randomization by hospital is appropriate to account for differences in clinical practice by hospital. In the present trial, randomization was not stratified by hospital. Sedation and analgesia protocols among hospitals did not obviously differ, and the randomization was insufficient due to small sample sizes in each participating hospital. This may introduce bias. Fifth, subjects who died before 24 hours after enrollment did not have data regarding their 24-hour lactate levels. For analysis in an intention-to-treat manner, we imputed the mean value of lactate, which could have influenced the outcome. Finally, we could not exclude the possibility of a selection bias. In our study, many patients were excluded after screening, which may have resulted in different results from retrospective studies.

In conclusion, the 24-hour lactate levels after drug infusion did not differ between the NMB (continuous infusion of rocuronium) and placebo groups. Furthermore, the continuous infusion of rocuronium for 24 hours did not improve survival or neurological outcomes at the time of hospital discharge. However, the rate of ICU-AW did not differ significantly between the two groups. This trial may be underpowered to detect clinical differences, and thus, we caution against using our findings to change clinical practice until a larger trial is conducted.

## Supporting information

S1 File CONSORT 2010 checklist. (DOC)

S2 File Continuous NMB—Study Protocol—English version. (DOCX)

S3 File Continuous NMB—Study Protocol—Korean version. (DOCX)

S4 File Raw data. (XLSX)
