# Supplementary material for: Continuous neuromuscular blockade infusion for out-of-hospital cardiac arrest patients treated with targeted temperature management: A multicenter randomized controlled trial
Source: PLoS One. 2018 Dec 17;13(12):e0209327. doi: 10.1371/journal.pone.0209327 (PMC6296517; doi:10.1371/journal.pone.0209327)
Supplement: S2 File — (DOCX) [file pone.0209327.s002.docx]

**Clinical Study Protocol**

**Title : Continuous neuromuscular blocking agent for out-of hospital cardiac arrest; Multicenter randomized controlled trial**

**Study Type : Prospective, Randomized Trial**

**Principle Investigator : Chun Song Youn**

**Seoul St. Mary’s Hospital, The Catholic University of Korea**

**ycs1005@catholic.ac.kr**

**Co-investigator**

**Byung Kook Lee, Chonnam National University Medical School**

**In Soo Cho, Hanil General Hospital, Korea Electric Power Medical Corporation, Seoul, Korea**

**Joo Suk Oh, Uijeongbu ST. Mary’s Hospital, The Catholic University of Korea Wook Jin Choi, Ulsan University College of Medicine, Ulsan, Korea**

**Jung Hee Wee, Yeouido ST. Mary’s Hospital, The Catholic University of Korea**

**Chang Sun Kim, Hanyang University of Korea, Guri, Korea**

**Won Young Kim, Asan Medical Center, University of Ulsan College of Medicine, Seoul, Korea**

**1. BACKGROUND**

**1.1 Epidemiology of cardiac arrest patients**

Out-of-hospital cardiac arrest (OHCA) occurs in more than 300,000 patients in the United States each year with an estimated mortality of greater than 90% [1,2]. In Korea, about 30,000 deaths from OHCA occur per year [3]. Post-cardiac arrest syndrome is characterized by a systemic inflammatory response that occurs after ischemic-reperfusion injury. In two randomized studies published in 2002, therapeutic hypothermia (TH) has been shown to improve survival and improve neurological prognosis in OHCA patients [4,5]. Recently, it has been recommended as standard therapy for patients with OHCA.

**1.2 Therapeutic hypothermia and NMB in post-cardiac arrest**

Neuromuscular blockers may be used for the purpose of preventing and treating shivering of patients during induction and maintenance of TH. However, neuromuscular blockers have the disadvantage of causing polyneuropathy and muscle weakness in ICU patients [6,7]. For this reason, the continuous infusion of neuromuscular blocking agents during TH in patients with OHCA remains controversial. Recently, however, Nielsen et al. performed a large, randomized, multicenter trial evaluating patients cooled to 33°C vs. a temperature at 36°C, and found no difference in survival or neurological outcome. The use of neuromuscular blockade (NMB) was minimized in both arms of the Nielsen et al. study [8]. In two randomized studies published in 2002, neuromuscular blocking agents were actively used, which is a major difference from Nielsen et al. This raises the question of whether differences in NMB usage in the two arms could have contributed to the difference in outcome that was not seen in the Nielsen et al. study.

**1.3 Neuromuscular Blockers in Other Critical Illness**

Papazian et al. published a randomized study of the effects of neuromuscular blockers in patients with early adult respiratory distress syndrome (ARDS). In this study, patients who received neuromuscular blockers showed an improvement in survival (41% vs 32%), no increase in myopathy [9]. Alhazzani et al. conducted a meta-analysis of three trials using cisatracurium early in ARDS, and found an overall mortality benefit and no increase in ICU-acquired myopathy. [10]. In addition, Steingrub et al. reported that neuromuscular blockers improve survival in patients with severe sepsis [11].

**1.4 Theoretical basis of neuromuscular blockers**

1) Reduced global oxygen consumption and metabolic demand: By reducing any voluntary or involuntary muscle activity (including potential shivering) NMB may lower global oxygen consumption even further, thereby preserving limited oxygen delivery for other vital organs including the brain [12]. This effect of NMB on oxygen consumption has been shown previously in patients with traumatic brain injury, respiratory failure, and after cardiac revascularization.

2) Improvement in pulmonary gas exchange: A previous randomized study in patients with ARDS found that continuous use of NMB for 48-hrs resulted in better oxygenation when compared to the placebo group [13]. This may occur due to increased chest wall compliance and a decrease in ventilator dyssynchrony.

3) Decreased inflammation: The continuous use of NMB for 48-hrs has previously been shown to decrease both pulmonary and systemic inflammation, specifically decreased levels of IL-1βand IL-6 [14]. We have previously shown that higher levels of IL-6 in the post-cardiac arrest patient are associated with increased mortality [15].

**1.5 Effects of Neuromuscular Blockers in Patients with OHCA**

The effect of neuromuscular blocking agents in patients with cardiac arrest was not well known. Patients who are in coma after resuscitation have similar systemic inflammatory responses to sepsis, resulting in increased metabolism, inadequate oxygen supply and consumption, resulting in lactic acidosis. Elevated serum lactate is associated with post-cardiac arrest mortality and neurological outcome [16,17]. Continuous neuromuscular blockers may lower the metabolic demand and reduce inflammatory markers, which may improve the patient's outcome. Salciccioli et al. reported that sustained neuromuscular blocking agents lowered the mortality rate of patients, but the reliability of the results is limited by retrospective analysis [18].

**2. STUDY OBJECTIVE**

The effects of neuromuscular blockers in patients with OHCA treated with TH are not yet clear. In this study, we compare the clinical effects of continuous neuromuscular blockers with control group.

**2.1 To determine if continuous NMB attenuates lactate levels in post-OHCA patients.**

We will perform a multicenter trial randomizing 80 patients to receive continuous NMB for 24-hrs after ROSC or usual care with the primary outcome of reduction in lactate levels. Lactate measurements will be obtained at time 0, 12 and 24-hrs.

**2.2 To determine if continuous NMB improves clinical outcomes in post-OHCA patients.**

We will compare a hierarchical clinical endpoint (including mortality, neurological outcome and length of stay) in patients randomized to receive NMB vs. usual care.

**3. STUDY DESIGN**

**3.1 Study Hypothesis**

: Continuous NMB administration improves the patient outcome.

**3.2 Design**

: Multicenter randomized, open-label, phase II trial in post-OHCA patients comparing sustained NMB administration for 24hrs to standard of care after ROSC.


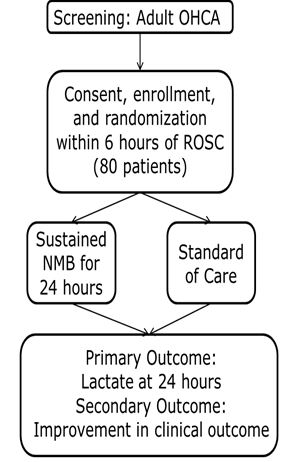


**3.3. Study Method**

1) shivering control

The appropriate analgesia and sedation should be performed first in both the intervention group and the control group. There is no restriction on the drugs required for analgesia and sedation. Shivering is judged by the following Bedside Shivering Assessment Scale (BSAS). If the BSAS score is 0, shivering control is deemed appropriate and additional control is required if the BSAS score is 2 or 3. If the BSAS score is 1, additional shivering control is performed based on clinical judgment.

Bedside Shivering Assessment Scale (BSAS)

| Score | Type | Location |
| --- | --- | --- |
| 0 | None | No shivering is detected on palpation of the masseter, neck, or chest muscles |
| 1 | Mild | Shivering is localized to the neck and thorax only |
| 2 | Moderate | Shivering involves gross movement of the upper extremities |
| 3 | Severe | Shivering involves gross movements of the trunk and upper and lower extremities |

2) Continuous NMB Group

Use rocuromium (esmeron) as a neuromuscular blocking agent.

Patients assigned to the NMB arm received a bolus of 0.6 mg/kg of rocuronium at the beginning of TTM, followed by an infusion of 0.3–0.6 mg/kg/hr.

Duration of injection: total 24 hours.

2) Control group

The same amount of normal saline as the intervention group is injected.

If analgesia and sedative agents do not provide shivering control, ie if NMB administration is

considered clinically necessary, 3 mg of rocuronium may be injected with bolus.

Specific indications are as follows

1. intractable shivering (BSAS score above 2 after administration of analgesia and sedative agents)
2. asynchrony with the mechanical ventilator not controlled with analgesia and sedative agents

**3.4. Concomitant medication**

Both groups use analgesic and sedative agents for shivering control. There is no restriction on the drugs required for analgesia and sedation.

**3.5. Prohibited drugs**

In this study, rocuronium is used as a neuromuscular blocking agent for continuous NMB group. Neuromuscular blocking agents other than rocuronium are not used.

**3.6 Neuromuscular blocking agent**

Neuromuscular blocking agents are classified into non-depolarizing blocking agents and depolarizing blocking agents. Typical examples of depolarizing blocking agents are succinylcholine. Succinylcholine is not suitable for use as a 24-hour continuous dose regimen due to its relatively short half-life. Among non-depolarizing blocking agents, rocuronium, cisatracurium, and atracurium are used in clinical practice in Korea. The reason for using rocuronium in this study is that rocuronium is most commonly used in participating organizations.

**4. STUDY ENDPOINTS**

**4.1. Efficacy Outcome**

Primary Outcome

- Serum lactate levels at 24 hrs

Secondary Outcome

- Lactate change over time

- In-hospital mortality

- Neurological outcome at hospital discharge

- Length of ICU stay

- changes of PaO_2_:FiO_2_ ratio

**4.2. Safety and feasibility outcome**

**4.2.1. feasibility endpoints**

1) total enrollment

2) time required to enroll and start study drug

3) extent of use of NMB in control arm

4) frequency of refusal of consent

**4.2.2safety endpoints**

1) muscle weakness : Use the Medical Research Council (MRC) scale. The score of each muscle is evaluated as 0 (paralysis) to 5 (normal strength) and the sum is between 0 and 60 points. ICU-acquired paresis is defined as MRC score of 48 points or less when leaving ICU [19].

2) Investigate all adverse events considered to be drug related. All reported adverse events (AEs) and all serious adverse events (SAEs) will be evaluated.

**5. Study duration**

The overall study is expected to include a two-month preparation period, including IRB approval, and a total study period of approximately 14 months, including 10 months of clinical trial enrollment, clinical follow-up, ending and analysis.

**6. RANDOM ALLOCATION SCHEDULE**

To maximize the comparability of the two groups and to ensure the scientific validity of the clinical trials, a randomization method will be implemented. Random assignments were assigned using block randomization without stratification and randomized assignments were completed by applying a block size of 2, 4, or 6 to maintain unpredictability, the basic principle of randomization.

The randomization of this trial will generate a random number using SAS Enterprise Guide 4 by an independent statistician not related to the study. Placement envelopes with random assignment numbers and placement groups should be blinded with opaque materials and should remain sealed until randomization is performed for each subject.

The person responsible for the clinical trial shall confirm the selection exclusion criteria and, if they are appropriate subjects, give a random assignment number, release the suture of the placement envelope and follow the procedure assigned to the subject. The person responsible for the clinical trial shall record release information (release date and release date) immediately upon unsealing of the placement envelope. In addition, a randomized envelope that has already been published once will not allow another subject to reassign the random assignment code even if the subject withdraws the consent.

**7. SUBJECT RECRUITMENT METHOD**

The patients who underwent targeted temperature management among OHCA patients will be prospectively recruited.

**7.1 Inclusion criteria**

1) Adult (19 years or older)

2) OHCA with sustained ROSC

3) Comatose (i.e., not following commands) following ROSC

4) Undergoing targeted temperature management (TTM)

5) Time of enrollment ≤ 6hrs from ROSC

**7.2 Exclusion criteria**

1) Pre-existing dementia, brain injury, or dependence on others for ADLs (CPC > 3)

2) Traumatic etiology for cardiac arrest

3) Protected population (pregnant, prisoner)

**7.3 Subjects selected**

Patients who have undergone targeted temperature management in OHCA are screened for clinical trial enrollment. If the patient complies with the registration criteria and a written consent is obtained within 6 hours, the patient will be enrolled in the trial.

**8. STOP AND DROP CRITERIA**

All subjects have the right to terminate their participation at any time during the clinical trial without infringing their rights.

- Medically necessary

Ex.) If serious illnesses develop irrespective of participation in clinical trials, and clinical trials are no longer considered to be the best option for the subject.

In this case, all of the subjects should be recorded regardless of completion of the clinical trial. In case of early withdrawal, the researcher must record the reason and report it immediately to the Data Coordinating Center (DCC). However, follow-up should be completed for all subjects, regardless of whether the assigned therapy is applied or not. The investigator should make every attempt to obtain follow-up information, except when the subject refuses to follow-up. Regardless of whether the assigned treatment is applied or not, all procedures related to the evaluation variable should be implemented.

**9. CLINICAL TRIAL SCHEDULE**

|  | Screening | (Random~F/U) | | | |  |  |
| --- | --- | --- | --- | --- | --- | --- | --- |
|  | < 6hrs after ROSC | 0hr after enrollment  (random) | 12hrs after enrollment | 24hrs after  enrollment | 36hrs after  enrollment | ICU discharge | Hospital discharge |
| Informed consent | X |  |  |  |  |  |  |
| Pregnancy test (urine)^1^ | X |  |  |  |  |  |  |
| Eligibility assessment |  | X |  |  |  |  |  |
| Demographics |  | X |  |  |  |  |  |
| Medical history |  | X |  |  |  |  |  |
| Vital sign |  | X |  |  |  |  |  |
| Neurologic exam. |  | X |  |  |  |  |  |
| SOFA^2^ |  | X |  |  |  |  |  |
| APACHE II^3^ |  | X |  |  |  |  |  |
| Laboratory finding |  | X |  |  |  |  |  |
| TTM^4^ |  | X | X | X |  |  |  |
| Drug |  | X | X | X |  |  |  |
| Lactate |  | X | X | X | X |  |  |
| Muscle weakness |  |  |  |  |  | X |  |
| Neurologic outcome |  |  |  |  |  |  | X |

1. Pre-menopausal women only.

2. SOFA : sequential organ failure assessment

3. APACHE II :Acute Physiology and Chronic Health Evaluation II

4. TTM : targeted temperature management

**10. STANDARD TREATMENT OF TARGET DISEASE**

Targeted temperature management is a standard treatment for patients with cardiac arrest and is recommended as Class I by the American Heart Association. Proper sedation is essential to prevent shivering during the induction and maintenance of hypothermia. In addition, shivering that is not controlled by the sedative agent should be controlled using the NM Blocker.

There are no standard guidelines for the use of NM blockers during TTM to date. As mentioned in 1.4 and 1.5, there are some retrospective studies that the use of NM blocker can theoretically improve the survival rate of OHCA patients and improve the survival rate, but it also has disadvantages of causing polyneuropathy and muscle weakness. Future research will need to be done on the use of NM blockers and the outcome of patients.

11. SAFETY OF SUBJECT

11.1 Informed consent

11.1.1 Contents of informed consent

A total of 80 subjects are enrolled in this trial. Do not enroll any person in a vulnerable environment (prisoners or persons with restrictions on follow-up observations in a protective facility). Pregnant women and children are excluded from this trial due to ethical and safety considerations. In addition, women who are likely to become pregnant should check their blood or urine pregnancy before registering to check for a negative.

Before collecting clinical trial data, all details of the clinical trial should be explained to the subject, including:

- This study is conducted for research purposes
- Participation in clinical trials is voluntary and there are no restrictions on dropouts
- What are the benefits to the subject when they participate?
- Potential Risks and Benefits
- Any contact you may contact at any time, the purpose of the clinical trial, the alternative treatment, the method of placement for treatment (random if applicable), and the ability to refuse or accept participation without affecting your future care

All types of data from clinical trials can be medical records, electrocardiograms, basic blood tests, and so on. These data can be obtained for clinical trial purposes and can also be obtained in general clinical practice.

**11.1.2 Acquisition of informed consent**

This is a study of comatose patients who recovered spontaneous circulation after an OHCA and informed consent can not be obtained from the patient directly. Therefore, the informed consent will be given to the patient's legal representative. In principle, no consent can be obtained if there is no legal representative. If you have more than one legal representative, you will be deemed to have obtained an informed consent. The consent form can not be obtained by telephone, and must be received in person. If the patient regains consciousness after treatment, he or she will receive informed consent once again after the patient has stabilized. Because the subject is unconscious, the legal representative agrees to participate in the clinical trial, but if the subject regains consciousness, he or she will get his informed consent again. At this time, participation in the clinical trial can be withdrawn, and the clinical data of the subject already collected will be automatically discarded.

11.2 Potential risk

The informed consent will be provided for IRB deliberation by each participating organization. This form describes the potential risks, protection from these risks and potential benefits based on knowledge from the past to the present. The informed consent must be obtained prior to randomized treatment.

11.2.1 Related risk

Related risk associated with medication

Neuromuscular blockers (Rocuronium) are currently in use. It can cause cardiovascular side effects, and anaphylaxis, but in most cases the benefits outweigh the risks.

11.2.2 Safety measures to prevent risk

The Clinical Events Committee (CEC) will closely monitor the risk of any potential clinical trial participants. All collected data will be regularly monitored for serious adverse events or death events related to the study. In case of a serious adverse reaction, the subject will be contacted by 24-hour contact, and the researcher who detects it will report serious adverse reaction to IRB and related authorities within 24 hours.

11.3 Adverse events / Serious adverse events

11.3.1adverse events

Once an adverse reaction is identified, the investigator should collect all the necessary information. In addition, subjects are instructed to contact the researcher or researcher to report any significant adverse events that occurred during the study. All adverse events should be tracked until a stable clinical condition is reached, and all treatments and results required for adverse events should be recorded.

**Adverse events severity**

The investigator will use the following definition to assess the severity of each adverse event.

- mild: Temporary signs or symptoms without sequelae that do not interfere with the patient's routine activities or are resolved without treatment
- moderate: Interfering with the patient's daily activities or requiring symptomatic treatment
- severe: Serious discomfort, significant impact on patient's daily activities, and need of treatment

**Determine the relationship between the adverse events and the test drug**

The investigator should determine the relationship between the adverse reaction and the test drug by applying the following criteria.

**Certain**

The relationship between the administration and use of medicines is reasonable and is not explained by other medicines, chemicals, or accompanying diseases. When the medicines are discontinued, they show a clinically valid response.

**Probable/Likely**

If the temporal relationship with the administration or use of medicines is reasonable and does not appear to be due to other medicines, chemicals or accompanying diseases. No re-administration information.

**Possible**

The time relationship between administration and use of medicines is reasonable, but it is also explained that the medicines are related to other medicines or chemicals or accompanying diseases. If the information on discontinuation of medicines is insufficient or unclear

**Unlikely**

It is a temporary case where it is unlikely that there will be a causal relationship between the use and use of medicines. Possible explanations for other medicines, chemicals or potential diseases

**Conditional/Unclassified**

If you need more information or are reviewing additional information for a proper evaluation

**Unassessible/Unclassifiable**

If information can not be judged as insufficient or conflicting, and can not be supplemented or confirmed

11.3.2 Serious adverse events

In case of a serious adverse event, the subject will be contacted by the 24-hour contact, and the investigator will report serious adverse reactions to the IRB and the authorities within 24 hours.

Critical adverse events are defined as one or more of the following:.

- Death or life threat
- Causes permanent impairment or deterioration
- Possible impairment, risk of life threat, or the need for medical intervention is required or the length of stay is extended

All significant adverse events should be followed up with or without sequelae until the response is resolved.

12. CONFIDENTIALITY

All information generated in this trial should be considered confidential and should not be disclosed to anyone not directly involved in the clinical trial without the written consent of the sponsor. All data used in the summary and analysis of this trial are anonymized and the identification of the subject is only possible with the clinical trial number assigned to the case record. Access to clinical trial subjects' data is only available to authorized personnel, researchers, and clinical trial personnel.

13. STATISTICAL ANALYSIS

13.1 Sample size calculation

The primary end point is the blood lactate level after 24 hours. In previous studies, the lactate levels of NMB (neuromuscular blockage) were 4.3 ± 3.8 and no-NMB group were 1.6 ± 1.0 mmol / L, respectively [19]. Assuming that the difference between the two groups with conservative estimates is 2.0 ± 3.15, the number of subjects required for the criterion of alpha = 0.05 and power = 80% is approximately 40 in each group.

13.2 Data analysis

**13.2.1 Lactate level**

In case of primary outcome (Lactate level at 24hr), two sample t-test or Wilcoxon rank sum test is performed according to normal distribution. In addition, the lactate level analysis method (0, 12hr, 24hr) according to each time point requires analysis of the covariance by time. After choosing the model values, we will select the appropriate model based on the Akaike Information Criteria (AIC) values in each model. The values of all models (= estimated values) are based on the REML (Restricted Maximum Likelihood Estimation) method.

**13.2.2 Secondary endpoint (mortality, neurological outcome, ICU length of stay, changes of PaO2:FiO2 ratio)**

Comparisons of categorical variables (mortality, neurologic outcome at hospital discharge) are performed using χ^2^ or Fisher exact tests, as appropriate. The Mann–Whitney *U* test or independent *t*-test is conducted for comparisons of continuous variables (ICU length of stay). A linear mixed model analysis was conducted to assess changes in lactate levels and changes in the PaO_2_:FiO_2_ ratio over time.

**13.2.3 Missing data**

In case of missing data for the primary outcome (i.e. patient dies within 24-hrs), we will impute a lactate value. This value will be the mean (or median as appropriate) lactate value at 24-hrs for those patients (both treatment and control arm) that subsequently died before hospital discharge. We do not expect to have missing data for the secondary outcome.

14. REGULATORY RESPONSIBILITY

14.1 Investigator’s responsibility

The investigator is responsible for ensuring that clinical trials are carried out in accordance with all signed agreements and protocols. Each researcher must also understand and sign the researcher agreement.

- Sign and follow the researcher agreement.
- Participate in researchers' meetings and training sessions as planned by the sponsor.
- The investigator should be willing to perform and have the ability to perform the treatment procedures as described in the clinical trial protocol.
- The researcher must comply with all the essential requirements of the protocol and provide appropriate data for the analysis
- Receive written informed consent from clinical trial subject before specific procedures are performed.
- If necessary, adjust the procedures of the hospital (unless the safety and well-being of the subject is compromised).
- Comply with the central analysis guidelines.

**15. RECORD**

Each researcher should maintain the following accurate, complete, and most recent records of clinical trials: Some of these records are available in computerized form in the DCC, but the final responsibility for maintaining the records remains with the researcher.

**16. REPORT**

The following is a report on the responsibility of the researcher. This table indicates to whom the report should be sent and to what interval or when. Some of these reports are developed with the help of DCC. However, the final responsibility of reporting is to the researcher.

**[Report required for researchers]**

| **Types of reports** | **Subject to be reported** | **Reporting period** |
| --- | --- | --- |
| Serious adverse events | IRB | According to local regulations |
|  | DCC | Within 7 working days in case of death / life threat |
|  |  | Otherwise within 15 working days |
| Interim report | IRB | According to local regulations |
|  |  |  |
|  |  |  |
| Violation of clinical trial plan | IRB | According to local regulations |
|  | DCC | Within 7 working days |
| Final summary report | DCC | Within 1 months |
|  |  |  |

**17. RECORD RETENTION**

The required documentation will be kept for at least three years after the publication of the clinical trial has officially ended and the final outcome report (including any type of paper or presentation, and no format restrictions) has been issued. Details of record preservation are in accordance with local regulations.

**18. 참고 문헌**

1. McNally B, Robb R, Mehta M, Vellano K, Valderrama AL, Yoon PW, Sasson C, Crouch A, Perez AB, Merritt R, Kellermann A, Centers for Disease C, Prevention. Out-of-hospital cardiac arrest surveillance --- cardiac arrest registry to enhance survival (cares), united states, october 1, 2005--december 31, 2010. Morbidity and mortality weekly report.Surveillance summaries. 2011;60:1-19

2. Lloyd-Jones D, Adams RJ, Brown TM, Carnethon M, Dai S, De Simone G, Ferguson TB, Ford E, Furie K, Gillespie C, Go A, Greenlund K, Haase N, Hailpern S, Ho PM, Howard V, Kissela B, Kittner S, Lackland D, Lisabeth L, Marelli A, McDermott MM, Meigs J, Mozaffarian D, Mussolino M, Nichol G, Roger VL, Rosamond W, Sacco R, Sorlie P, Thom T, Wasserthiel-Smoller S, Wong ND, Wylie-Rosett J. Heart disease and stroke statistics--2010 update: A report from the american heart association. Circulation. 2010;121:e46-e215

3. National Center for Disease Control

4. Group HaCAS. Mild therapeutic hypothermia to improve the neurologic outcome after cardiac arrest.The New England journal of medicine. 2002;346:549-556

5. Bernard SA, Gray TW, Buist MD, Jones BM, Silvester W, Gutteridge G, Smith K. Treatment of comatose survivors of out-of-hospital cardiac arrest with induced hypothermia. The New England journal of medicine. 2002;346:557-563

6. Gooch JL, Suchyta MR, Balbierz JM, Petajan JH, Clemmer TP. Prolonged paralysis after treatment with neuromuscular junction blocking agents. Critical care medicine. 1991;19:1125-1131

7. Segredo V, Caldwell JE, Matthay MA, Sharma ML, Gruenke LD, Miller RD. Persistent paralysis in critically ill patients after long-term administration of vecuronium. The New England journal of medicine. 1992;327:524-528

8. Nielsen N, Wetterslev J, Cronberg T, Erlinge D, Gasche Y, Hassager C, Horn J, Hovdenes J, Kjaergaard J, Kuiper M, Pellis T, Stammet P, Wanscher M, Wise MP, Aneman A, Al-Subaie N, Boesgaard S, Bro-Jeppesen J, Brunetti I, Bugge JF, Hingston CD, Juffermans NP, Koopmans M, Kober L, Langorgen J, Lilja G, Moller JE, Rundgren M, Rylander C, Smid O, Werer C, Winkel P, Friberg H. Targeted temperature management at 33 degrees c versus 36 degrees c after cardiac arrest. The New England journal of medicine. 2013

9. Papazian L, Forel JM, Gacouin A, Penot-Ragon C, Perrin G, Loundou A, Jaber S, Arnal JM, Perez D, Seghboyan JM, Constantin JM, Courant P, Lefrant JY, Guerin C, Prat G, Morange S, Roch A. Neuromuscular blockers in early acute respiratory distress syndrome. The New England journal of medicine. 2010;363:1107-1116

10. Alhazzani W, Alshahrani M, Jaeschke R, Forel JM, Papazian L, Sevransky J, Meade MO.Neuromuscular blocking agents in acute respiratory distress syndrome: A systematic review and meta-analysis of randomized controlled trials. Crit Care. 2013;17:R43

11. Steingrub JS, Lagu T, Rothberg MB, Nathanson BH, Raghunathan K, Lindenauer PK. Treatment with neuromuscular blocking agents and the risk of in-hospital mortality among mechanically ventilated patients with severe sepsis. Critical care medicine. 2013

12. Manthous CA, Hall JB, Kushner R, Schmidt GA, Russo G, Wood LD. The effect of mechanical ventilation on oxygen consumption in critically ill patients.American journal of respiratory and critical care medicine. 1995;151:210-214

13. Gainnier M, Roch A, Forel JM, Thirion X, Arnal JM, Donati S, Papazian L. Effect of neuromuscular blocking agents on gas exchange in patients presenting with acute respiratory distress syndrome. Critical care medicine. 2004;32:113-119

14. Forel JM, Roch A, Marin V, Michelet P, Demory D, Blache JL, Perrin G, Gainnier M, Bongrand P, Papazian L. Neuromuscular blocking agents decrease inflammatory response in patients presenting with acute respiratory distress syndrome. Critical care medicine. 2006;34:2749-2757

15. Peberdy; MA, Ornato; JP, Helm; SL, Thacker; LR, Callaway; CW, Rittenberger; JC, Cocchi; MN, Gaieski; DF, Abella; BS, Donnino; MW, Investigators ftNPARC. Abstract 239: Initial cytokine levels are associated with outcome after cardiac arrest. Circulation. 2013:128:A239

16. Cocchi MN, Miller J, Hunziker S, Carney E, Salciccioli J, Farris S, Joyce N, Zimetbaum P, Howell MD, Donnino MW. The association of lactate and vasopressor need for mortality prediction in survivors of cardiac arrest.Minervaanestesiologica. 2011;77:1063-1071

17. Donnino MW, Miller J, Goyal N, Loomba M, Sankey SS, Dolcourt B, Sherwin R, Otero R, Wira C. Effective lactate clearance is associated with improved outcome in post-cardiac arrest patients. Resuscitation. 2007;75:229-234

18. Salciccioli JD, Cocchi MN, Rittenberger JC, Peberdy MA, Ornato JP, Abella BS, Gaieski DF, Clore J, Gautam S, Giberson T, Callaway CW, Donnino MW. Continuous neuromuscular blockade is associated with decreased mortality in post-cardiac arrest patients. Resuscitation. 2013;84:1728-1733

19. De Jonghe B, Sharshar T, Lefaucheur JP, Authier FJ, Durand-Zaleski I, Boussarsar M, Cerf C, Renaud E, Mesrati F, Carlet J, Raphael JC, Outin H, Bastuji-Garin S, Groupe de Reflexion et d'Etude des Neuromyopathies en R. Paresis acquired in the intensive care unit: A prospective multicenter study. JAMA : the journal of the American Medical Association. 2002;288:2859-2867
