# Supplementary material for: Continuous neuromuscular blockade infusion for out-of-hospital cardiac arrest patients treated with targeted temperature management: A multicenter randomized controlled trial
Source: PLoS One. 2018 Dec 17;13(12):e0209327. doi: 10.1371/journal.pone.0209327 (PMC6296517; doi:10.1371/journal.pone.0209327)
Supplement: S3 File — (DOCX) [file pone.0209327.s003.docx]

**Continuous NM Blocker 연구**

**연구제목**

**국문 :심정지 환자에서 신경근육차단제의 효과, 무작위배정 다기관 연구**

**영문 : Continuous neuromuscular blocking agent for out-of hospital cardiac arrest; Multicenter randomized controlled trial**

**단계 : 학술용 / 연구자 주도 임상연구**

**연구기관 및 책임연구자 (소속과/직위/성명)**

**가톨릭대학교 서울성모병원 응급의학과 / 조교수 / 윤준성**

**서울특별시 서초구 반포대로 222 가톨릭대학교 서울성모병원**

**임상시험 실시 기관명 및 주소**

1. 서울 성모병원, 서울특별시 서초구 [반포대로](http://www.juso.go.kr/support/AddressMainSearch.do?searchType=TOTAL#self) 222

2. 여의도 성모병원, 서울특별시 영등포구 [63로](http://www.juso.go.kr/support/AddressMainSearch.do?searchType=TOTAL#self) 10

3. 의정부 성모병원, 경기도 의정부시 [천보로](http://www.juso.go.kr/support/AddressMainSearch.do?searchType=TOTAL#self) 271

4. 아산병원, 서울특별시 송파구 [강동대로](http://www.juso.go.kr/support/AddressMainSearch.do?searchType=TOTAL#self) 13

5. 전남대 병원, 광주광역시 동구 [제봉로](http://www.juso.go.kr/support/AddressMainSearch.do?searchType=TOTAL#self) 42

6. 한일병원, 서울특별시 도봉구 [우이천로](http://www.juso.go.kr/support/AddressMainSearch.do?searchType=TOTAL#self) 308

7. 한양대 구리병원, 경기도 구리시 [경춘로](http://www.juso.go.kr/support/AddressMainSearch.do?searchType=TOTAL#self) 153

8. 울산대 병원, 울산광역시 동구 [방어진순환도로](http://www.juso.go.kr/support/AddressMainSearch.do?searchType=TOTAL#self) 877

**기관별 연구책임자**

| **번호** | **성명** | **소속** | **직위** | **연락처(휴대폰)** |
| --- | --- | --- | --- | --- |
| 1 | 윤준성 | 서울성모병원 | 조교수 | 010-2480-9727 |
| 2 | 위정희 | 여의도성모병원 | 조교수 |  |
| 3 | 오주석 | 의정부성모병원 | 부교수 |  |
| 4 | 김원영 | 아산병원 | 부교수 |  |
| 5 | 이병국 | 전남대병원 | 조교수 |  |
| 6 | 조인수 | 한일병원 | 과장 |  |
| 7 | 김창선 | 한양대 구리병원 | 조교수 |  |
| 8 | 최욱진 | 울산대병원 | 조교수 |  |

**연구비 지원 기관**

2016년 성의장학 학술연구비

**1. 연구 배경**

**1.1 병원외심정지 환자의 역학**

병원외심정지는 미국에서 연간 300,000명 이상 발생하여 90% 이상의 사망률을 보이며 [1,2] 국내에서는 연간 30,000명가량 발생하며 95% 이상의 환자가 사망한다 [3]

심정지 후 증후군(post-cardiac arrest syndrome)은 ischemic-reperfusion injury후에 발생하는 전신의 염증반응이 특징적이다. 2002년에 발표된 2개의 무작위 배정연구에서 저체온 치료(therapeutic hypothermia)는 병원외심정지 환자의 생존률을 높여주며 신경학적 예후를 개선시키는 것으로 나타났고 [4,5] 최근에는 심정지 환자의 표준치료로 권고되고 있다.

**1.2 저체온 치료와 신경근육차단제**

저체온 치료를 유도 및 유지하는 과정에서 환자의 떨림(shivering)을 예방하고 치료하기 위한 목적으로 신경근육차단제가 사용될 수 있다. 하지만 신경근육차단제는 중환자에게서 polyneuropathy 및 muscle weakness를 유발할 수 있다는 단점이 있다 [6,7]. 이러한 이유로 병원외심정지 환자의 저체온 치료 도중 신경근육차단제의 지속적 사용은 논란의 여지가 있다. Nielsen등에 의한 대규모 다기관, 무작위 배정연구에서 병원외심정지 환자에게 목표체온을 33℃와 36℃로 달리 적용했을 때 생존률 및 신경학적 예후에 차이가 없다고 발표하였는데 이 대규모 연구에서는 신경근육차단제가 양군에서 모두 제한적으로 사용되었다 [8]. 2002년에 발표된 2개의 무작위 배정연구에서는 신경근육차단제가 적극적으로 사용되었으며 이는 Nielsen등의 연구와 큰 차이점이다. 연구 결과가 상이한 이유로, 밝혀지지 않은 교란요인인 신경근육차단제의 효과를 배제할 수 없다.

**1.3 다른 중환자 치료영역에서 신경근육차단제**

Papazian등은 초기 성인호흡곤란증후군(ARDS)환자에서 신경근육차단제의 효과에 대한 무작위 연구를 발표하였다. 이 연구에서 신경근육차단제를 투여받은 환자는 생존률의 향상 (41% vs 32%)을 보였으며 myopathy의 증가를 보이지 않았고 또한 인공호흡기를 부착하지 않은 기간에서도 향상된 결과(p < 0.05)를 보였다 [9]. 초기 ARDS환자에서 신경근육차단제의 효과에 대한 Alhazzani등의 메타분석에서도 myopathy의 증가 없이 생존률의 향상을 보였다 [10]. 또한, Steingrub등은 중증 패혈증환자에서 신경근육차단제가 생존률을 향상시킨다고 보고하였다 [11].

**1.4 신경근육차단제의 이론적 근거**

1) oxygen consumption 및 metabolic demand 감소 : muscle activity를 줄여줌으로 인해 oxygen consumption을 감소시켜준다. 또한 이로 인해 brain을 비롯한 다른 중요한 기관에 사소 공급을 늘릴 수 있다 [12].

2) pulmonary gas exchange 향상 : ARDS 환자를 대상으로한 연구에서 지속적인 신경근육차단제를 사용한 군에서 oxygenation의 향상을 보였다 [13]. 이는 주로 chest wall compliance 증가와 ventilator dyssynchrony의 감소로 인한다.

3) inflammation 감소 : 신경근육차단제의 지속적인 사용은 전신의 염증 반응의 감소를 가져온다. 이는 IL-1B와 IL-6의 감소로 나타난다 [14]. 특히 혈중 IL-6 농도는 병원외심정지 환자에서 사망률과 관계있다 [15].

**1.5 병원외심정지 환자에서 신경근육차단제의 효과**

병원외심정지 환자에서 신경근육차단제의 효과는 아직까지 잘 알려지지 않았다. 소생술 후 혼수상태에 있는 환자는 패혈증과 비슷한 전신의 염증반응을 보이며 metabolism의 증가, 부적절한 산소의 공급 및 소모등을 일으켜 결과적으로 lactic acidosis를 일으킨다. 혈중 lactate의 상승은 심정지 후 사망률 및 신경학적 예후와 연관있다 [16,17]. 지속적인 신경근육차단제가 metabolic demand를 낮추고 inflammatory marker를 감소시킨다면 이는 환자의 예후를 향상시킬 수 있다. Salciccioli등은 지속적인 신경근육차단제가 환자의 사망률은 낮춘다는 보고를 하였으나 이는 후향적 분석으로 결과의 신뢰도에 한계가 있다 [18].

**2. 연구 목적**

저체온 치료를 시행 받는 병원외 심정지 환자에서 신경근육차단제의 효과는 아직 명확히 알려지지 않았다. 이 연구에서는 신경근육차단제의 지속적 투여군과 그렇지 않은 군의 임상 효과를 비교한다.

**2.1 To determine if continuous NMB attenuates lactate levels in post-OHCA patients.**

: 80명의 환자를 대상으로 다기관 무작위 배정연구를 할 것이다. primary outcome은 24시간째 혈중 lactate 농도이며 혈중 lactate 농도는 0, 12, 24hr에 측정할 것이다.

**2.2 To determine if continuous NMB improves clinical outcomes in post-OHCA patients.**

: secondary outcome으로 여러 clinical endpoint를 비교할 것이다. 사망률, 퇴원시 신경학적 상태, ICU 재원기간등을 비교할 것이다.

**3. 연구 설계**

**3.1 연구 가설**

: 지속적인 NMB 투여가 환자의 예후를 향상시킨다.

**3.2 연구 설계**

: Multicenter randomized, open-label, phase II trial in post-OHCA patients comparing sustained NMB administration for 24hrs to standard of care after ROSC.


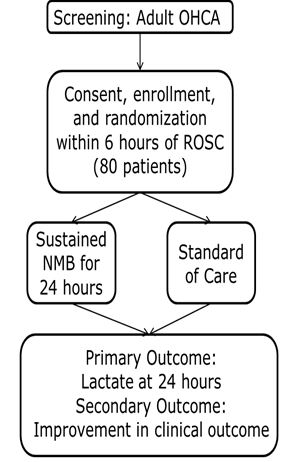


**3.3. 연구 방법**

1) shivering control

: Intervention group과 Control group 모두 적절한 analgesia and sedation을 먼저 시행한다. Analgesia and sedation에 필요한 약물에 대한 제한은 없다. Shivering은 다음의 도구로 판단한다. BSAS score 0이면 shivering control이 적절하다고 판단하고 BSAS score 2 혹은 3인 경우 추가적인 조절이 필요하다. BSAS score 1인 경우 임상적으로 판단하여 추가 shivering control을 시행한다.

Bedside Shivering Assessment Scale (BSAS)

| Score | Type | Location |
| --- | --- | --- |
| 0 | None | No shivering is detected on palpation of the masseter, neck, or chest muscles |
| 1 | Mild | Shivering is localized to the neck and thorax only |
| 2 | Moderate | Shivering involves gross movement of the upper extremities |
| 3 | Severe | Shivering involves gross movements of the trunk and upper and lower extremities |

2) 치료군 (NMB Group)

: Neuromuscular blocking agent는 rocuromium(esmeron)을 사용한다.

:rocuronium을 bolus로 0.6mg/kg 주입하고 이후 0.3 – 0.6 mg/kg/hr의 속도로 주입한다.

: 투입 지속 시간: 총 24시간동안 주입한다.

2) 대조군

: Intervention group과 같은 양의 normal saline을 주입한다.

: analgesia and sedative agent만으로 shivering control이 되지 않는 경우 즉, NMB의 투여가 임상적으로 필요하다고 판단되면 투여할 수 있고, 3mg을 bolus로 주사한다. 구체적인 적응증은 다음과 같다.

1. Intractable shivering (analgesics, sedative agent투여 후 BSAS Score 2이상의 shivering이 관찰될 경우)
2. Asynchrony with the mechanical ventilator (analgesics, sedative agent투여 후에도 관찰되는 경우)

**3.4. 병용 약물**

양군 모두 shivering control에 필요한 analgesic and sedative agent를 사용한다. 이 때 analgesia and sedation에 필요한 약물에 대한 제한은 없고 각 기관의 프로토콜에 따른다.

**3.5. 병용 금지 약물**

본 연구에서 치료군(continuous NMB group) 에서 사용되는 신경근육차단제는 각 기관 모두 rocuronium을 사용한다. Rocuronium 이외의 신경근육차단제는 사용하지 않는다.

**3.6. 신경근육차단제**

신경근육차단제는 크게 Non-depolarizing blocking agent와 Depolarizing blocking agent로 나뉘며 depolarizing blocking agent는 대표적으로 succinylcholine이 있다. Succinylcholine은 비교적 짧은 반감기로 인해 24시간 지속투여 요법으로 사용하기에는 적절하지 않다. Non-depolarizing blocking agent중 현재 국내 임상에서는 rocuronium, cisatracurium, atracurium등이 사용되고 있으며 효과나 부작용등에 큰 차이는 없다. 본 연구에서 rocuronium을 사용하는 이유는 본 연구에 참여하는 기관에서 가장 많이 사용되고 있기 때문이다.

**4. 평가 변수**

**4.1. 유효성 평가 기준**

일차 평가 변수

- 24시간째 혈중 Lactate level

이차 평가 변수

- Lactate change over time

-In-hospital mortality

- Neurological outcome at hospital discharge

- Length of ICU stay

- changes of PaO2:FiO2 ratio

**4.2. 안전성 평가 기준**

**4.2.1feasibility endpoints**

1) total enrollment

2) time required to enroll and start study drug

3) extent of use of NMB in control arm

4) frequency of refusal of consent

**4.2.2safety endpoints**

1) muscle weakness : Medical Research Council (MRC) scale을 이용한다. 각 근육의 점수는 0 (paralysis) ~ 5 (normal strength)로 평가하며 총합은 0~60점 사이이다. ICU-acquired paresis는 ICU 퇴실시에 MRC score 48점 이하로 정의된다 [19].

2) 약물과 관련있다고 여겨지는 모든 adverse event를 조사한다.보고되는 모든 이상반응(Adverse Events, AEs)과 중대 부작용들(all Serious Adverse Events, SAEs)등을 통하여 평가될 것이다.

**5. 진행 기간**

전반적 연구는 IRB 승인 등 2개월간의 준비과정을 포함하여, 10개월간의 임상시험 대상자 등록, 임상추적 관찰기간과 종료 및 분석 기간을 포함하여 약 14개월간의 총 연구기간이 예상된다.

**6. 무작위 배정 계획 (Random allocation schedule) 및 운영**

두 군의 비교성을 극대화하고, 각 군의 배정에 연구진의 주관이 개입되지 않도록 함으로써 임상시험의 과학적 타당성을 보장하고자 확률론적 이론에 의한 배정 방법인 무작위배정법을 시행할 예정이다. 무작위배정은 층화없이 블록 무작위 배정법 (Block randomization)을 이용하여 배정하였으며, 무작위화의 기본 원칙인 예측 불가능(Unpredictability)을 유지하기 위하여 block size를 2 또는 4 또는 6으로 적용하여 무작위 배정표를 완성할 것이다.

본 임상시험의 무작위 배정은 연구와 관련이 없는 독립된 통계학자가 SAS Enterprise Guide 4를 이용하여 난수를 발생시킬 것이다. 무작위 배정 번호와 배정군이 기록된 배정봉투는 불투명한 재질로 하여 맹검이 될 수 있도록 하며, 피험자 별로 무작위 배정이 실시될 때까지 봉합된 상태를 유지하도록 한다.

임상시험 책임자 또는 임상시험담당자는 선정제외기준을 최종 확인하여 적합한 피험자인 경우, 무작위 배정 번호를 부여하고, 배정봉투의 봉합을 해제하여 해당 피험자에게 배정된 방법으로 시술한다. 임상시험책임자 또는 임상시험 담당자는 배정봉투의 봉합 해제 즉시 해제정보(해제일과 해제자)를 기록해야 한다. 또한, 이미 한번 공개된 무작위 배정 봉투는 피험자가 동의를 철회하였다 하더라도 다른 피험자에게 해당 무작위 배정코드를 재부여 할 수 없게 할 것이다.

**7. 피험자 모집 방법**

병원외심정지 환자 중 저체온 치료를 시행 받는 환자를 대상으로 피험자 선정 제외 기준에 부합되는 환자를 전향적으로 모집할 예정이다.

**7.1 피험자 선정 기준**

1) Adult (19세 이상)

2) OHCA with sustained ROSC

3) Comatose (i.e., not following commands) following ROSC

4) Undergoing targeted temperature management (TTM)

5) Time of enrollment ≤ 6hrs from ROSC

**7.2 피험자 제외 기준**

1) Pre-existing dementia, brain injury, or dependence on others for ADLs (CPC > 3)

2) Traumatic etiology for cardiac arrest

3) Protected population (pregnant, prisoner)

**7.3 대상자 선정**

병원외심정지 환자 중 저체온 치료를 시행받기로 한 환자는 임상시험 등록을 위한 스크리닝을 받는다. 만약 등록기준에 적합하고 6시간 이내에 서면동의서가 취득되었다면 해당 환자는 본 임상시험에 등록된다.

**8. 중지 및 탈락 기준**

모든 대상자는 임상시험 기간 동안 어떠한 시점에서라도 권리를 침해 받지 않고 참여를 중지할 수 있는 권리가 있다.

- 의학적으로 필요한 경우

예) 임상시험 참여와 관계없는 중대한 질환이 발생함, 임상시험이 더 이상 대상자에게 최선의 방법이 아니라고 판단될 경우

이러한 경우 각 대상자의 임상시험 완료에 상관없이 모두 기록되어야 한다. 만약 조기 탈락한 경우 연구자는 그 이유를 기록하고 바로 DCC(Data coordinating Center, 데이터 협력 센터, 이하 DCC)로 보고하여야 한다. 하지만 모든 대상자에 있어 배정된 치료의 적용여부에 상관없이 추적관찰은 완료되어야 한다. 대상자가 추적관찰을 거부한 경우를 제외하고 연구자는 추적 관찰 정보를 얻기 위한 모든 시도를 해야 한다. 또한 배정된 치료의 적용여부에 상관없이 평가변수와 관련된 모든 절차는 이행되어야 한다.

**9. 임상시험 수행 일정표**

|  | Screening | (Random~F/U) | | | |  |  |
| --- | --- | --- | --- | --- | --- | --- | --- |
|  | < 6hrs after ROSC | 0hr after enrollment  (random) | 12hrs after enrollment | 24hrs after  enrollment | 36hrs after  enrollment | ICU discharge | Hospital discharge |
| Informed consent | X |  |  |  |  |  |  |
| Pregnancy test (urine)^1^ | X |  |  |  |  |  |  |
| Eligibility assessment |  | X |  |  |  |  |  |
| Demographics |  | X |  |  |  |  |  |
| Medical history |  | X |  |  |  |  |  |
| Vital sign |  | X |  |  |  |  |  |
| Neurologic exam. |  | X |  |  |  |  |  |
| SOFA^2^ |  | X |  |  |  |  |  |
| APACHE II^3^ |  | X |  |  |  |  |  |
| Laboratory finding |  | X |  |  |  |  |  |
| TTM^4^ |  | X | X | X |  |  |  |
| Drug |  | X | X | X |  |  |  |
| Lactate |  | X | X | X | X |  |  |
| Muscle weakness |  |  |  |  |  | X |  |
| Neurologic outcome |  |  |  |  |  |  | X |

1. 폐경전 여성에만 실시한다.

2. SOFA : sequential organ failure assessment

3. APACHE II :Acute Physiology and Chronic Health Evaluation II

4. TTM : targeted temperature management

**10. 대상 질환의 표준 치료법**

자발순환을 회복한 병원외심정지 환자에 대해 저체온 치료는 표준치료이며 미국심장학회에서 Class I으로 권고되고 있다. 저체온을 유도하고 유지하는 과정에서 shivering을 막기 위해 적절한 sedation은 필수적이다. 또한, sedative agent로 조절되지 않는 shivering은 NM Blocker를 사용하여 조절해야 한다.

현재까지 저체온 치료 도중 NM Blocker의 사용에 관한 표준 지침은 없다. 1.4, 1.5에서 언급했듯이 NM Blocker의 사용은 이론적으로 병원외심정지 환자의 생존률 향상에 도움이 될 수 있고, 환자의 생존률을 향상시킨다는 몇몇 후향적 연구가 있으나 polyneuropathy 및 muscle weakness를 유발할 수 있다는 단점 또한 가지고 있다. 향후 NM Blocker 사용과 환자의 예후에 관한 대규모 연구가 필요하다.

11. 대상자의 안전

11.1동의서

11.1.1 동의서의 내용

본 임상시험에서는 총 80명의 대상자를 등록한다. 취약한 환경에 있는 대상자(수감자 또는 보호시설에 있어 추적관찰에 제한이 있는 자)는 등록하지 않는다. 임신한 여성과 어린이는 윤리와 안전을 고려하여 본 임상시험에서는 제외된다. 또한 임신가능성이 있는 여성은 등록 전에 반드시 혈액 또는 소변 임신검사를 시행하여 음성을 확인하여야 한다.

임상시험 자료를 수집하기 전에 임상시험에 대한 모든 상세한 사항은 다음을 포함하여 대상자에게 설명되어야 한다:

- 본 임상시험은 연구 목적으로 수행된다는 점
- 임상시험 참여는 자발적이며 중도탈락에 어떠한 제한도 없다는 점
- 참여하였을 때 대상자에게 어떠한 이익이 있는지
- 잠재적인 위험과 이득
- 언제든지 연락할 수 있는 연락처, 임상시험의 목적, 대안 치료, 치료에 배정받는 (해당될 경우 무작위) 방법, 향후 진료에 어떠한 영향 없이 참여를 거절하거나 수락할 수 있다는 점

임상시험의 모든 종류의 자료는 의무기록, 심전도, 기본 혈액검사 등이 될 수 있다. 이러한 자료는 임상시험 목적으로 수행되어 얻어질 수 있고 또한 일반적인 임상에서의 진료에서도 얻어질 수 있다.

**11.1.2 동의서의 취득**

본 임상시험은 병원외심정지 후 자발순환을 회복한 혼수환자를 대상으로 하는 연구로 대상 환자의 특성상 환자 본인에게 동의서를 취득할 수 없다. 따라서 동의서는 환자의 법정 대리인에게 받을 것이다. 법정 대리인이 없는 경우는 원칙적으로 동의서를 취득 할 수 없다. 법정 대리인 1인 이상의 동의가 있는 경우 동의서를 취득한 것으로 인정한다. 동의서는 유선으로 취득할 수 없으며 반드시 대면으로 설명한 후 받아야 한다. 치료 후 환자가 의식을 회복한 경우 환자 상태가 안정화된 이후에 환자에게 직접 다시 한번 서면 동의를 받는다. 대상자가 의식이 없었던 관계로 법정 대리인이 임상시험 참여에 동의를 하였으나 대상자가 의식을 회복한 경우 직접 다시 한번 서면 동의를 받는다. 이때 임상시험 참여를 중도 철회할 수 있으며 이러한 경우 이미 수집된 대상자의 임상 자료는 자동 폐기된다.

11.2잠재적 위험

각 참여기관의 IRB 심의를 위하여 설명문 및 동의서 양식이 제공될 것이다. 이 양식에 잠재적 위험, 과거부터 현재를 아우른 지식을 기반으로 이러한 위험으로부터의 보호와 잠재적 이득에 대해 기술되어 있다. 대상자 설명문 및 동의서는 무작위 배정된 치료가 시행되기 전에 반드시 취득되어야 한다.

11.2.1관련된 위험성

약물 치료와 관련된 위험성

신경근육차단제(Rocuronium)은현재 많이 쓰이고 있다. 심혈관계 부작용, anaphylaxis등을 일으킬 수 있으나 대부분의 경우 이득이 위험을 상회한다.

11.2.2위험요소 방지를 위한 안전대책

DCC, CEC(Clinical Events Committee, 임상사건 검토 위원회, 이하 CEC)를 두고 임상시험에 참여하는 임상시험 대상자에게 발생가능성이 있는 위험요소가 있는지 엄격히 모니터 할것이다. 수집된 모든 데이터는 연구와 관련된 중대한 이상반응이나 사망사건 발생 여부에 대해 정기적으로 모니터 될 것이다. 중대한이상반응에대하여대상자에게교육하고, 중대한이상반응발생시대상자는 24시간연락처로연락하게되며, 이를감지한연구자는 24시간내에 IRB 및관계당국에중대한이상반응을보고한다.

11.3이상반응 / 중대한 이상반응

11.3.1이상반응

이상반응 발생이 확인되면 연구자는 필요한 모든 정보를 수집해야 한다. 뿐만 아니라 임상시험 대상자들은 연구자나 연구 담당자에게 연락해 연구 기간 사이에 발생한 모든 중요한 이상반응을 보고하도록 교육을 받는다. 모든 이상반응들은 해결책을 찾거나 안정적인 임상적 상태가 될 때까지 추적 관찰하며 이상반응에 필요한 모든 치료법과 그 결과는 기록되어야 한다.

**이상반응 중증도**

연구자는 다음의 정의를 이용하여 각 이상반응의 중증도를 평가할 것이다.

- 경증: 환자의 일상적이 활동을 방해하지 않거나 치료 없이 해결되는 후유증 없는 일시적인 징후나 증상의 인식
- 중증도: 환자의 일상적인 활동을 방해하거나 증상의 치료가 필요함
- 중증: 심각한 불편을 초래하고 환자의 일상적인 활동에 상당한 영향을 주며 치료가 필요함

**이상반응과 시험약과의 관계 판단**

연구자는 이상반응과 시험약과의 관계 판단을 해야 하며 다음의 기준을 적용하여 판단한다.

**확실함(certain)**

의약품 등의 투여․사용과의 전후 관계가 타당하고 다른 의약품이나 화학물질 또는 수반하는 질환으로 설명되지 아니하며, 그 의약품 등의 투여 중단 시 임상적으로 타당한 반응을 보이고, 필요에 따른 그 의약품 등의 재투여시, 약물학적 또는 현상학적으로 결정적인 경우

**상당히 확실함(probable/likely)**

의약품 등의 투여․사용과의 시간적 관계가 합당하고 다른 의약품이나 화학물질 또는 수반하는 질환에 따른 것으로 보이지 아니하며, 그 의약품 등의 투여 중단 시 임상적으로 합당한 반응을 보이는 경우(재투여 정보 없음)

**가능함(possible)**

의약품 등의 투여․사용과의 시간적 관계가 합당하나 다른 의약품이나 화학물질 또는 수반하는 질환에 따른 것으로도 설명되며, 그 의약품 등의 투여중단에 관한 정보가 부족하거나 불명확한 경우

**가능성 적음(unlikely)**

의약품 등의 투여․사용과 인과관계가 있을 것 같지 않은 일시적 사례이고, 다른 의약품이나 화학물질 또는 잠재된 질환에 따른 것으로도 타당한 설명이 가능한 경우

**평가 곤란(conditional/unclassified)**

적정한 평가를 위해 더 많은 자료가 필요하거나 추가 자료를 검토중인 경우

**평가 불가(unassessible/unclassifiable)**

정보가 불충분하거나 상충되어 판단할 수 없고 이를 보완하거나 확인할 수 없는 경우

11.3.2 중대한 이상반응

중대한이상반응발생시대상자는 24시간연락처로연락하게되며, 이를감지한연구자는 24시간내에 IRB 및관계당국에중대한이상반응을보고한다. 연구자는 윤리위원회/국내 관련 규정에 따라 임상시험 대상자에서 발생한 중대한 이상반응을 보고하여야 한다.

중대한 이상반응이란 아래의 내용 중 하나 또는 하나 이상을 만족하는 경우로 정의한다.

- 사망 또는 생명의 위협
- 영구적인 장애나 기능저하의 초래
- 잠재적 장애 가능성, 생명의 위험, 또는 의학적 중재의 필요로 입원을 요하거나 입원 기간이 연장된 경우

모든 중대한 이상반응은 반응이 해결될 때까지 후유증 유무 여부 상관없이 추적관찰이 이루어져야 한다.

중대한 이상반응과 중증의 이상반응은 구별되어야 한다. 중증의 이상반응은 중대한 이상반응이 아닐 수 있으며, 중대한 이상반응은 중증인 것으로 간주될 필요는 없다. “중증의” 이 용어는 어떤 사건의 중증도(경증, 중등도, 중증)를 기술한다. 예를 들어 중증의 두통인 경우 사건 그 자체는 사소한 의료적 상황(의미)일 수 있다. 이것은 “중대한”과 동일하지 않다. “중대한” 은 일반적으로 환자의 생명이나 기능에 위협이 가하는 사건에 대한 환자/사건의 결과 또는 그 행동 기준에 기초한다.

12. 비밀 유지

본 임상시험에서 발생한 모든 정보는 기밀로 간주되어야 하며 의뢰자의 서면 동의 없이는 임상시험에 직접 관련되지 않은 어떠한 사람에게도 공개되어서는 안 된다. 본 임상시험의 요약과 분석에 사용된 모든 자료는 익명화되어 임상시험 대상자의 식별은 오직 증례 기록서에 부여된 임상시험번호로만 가능하다. 임상시험 대상자의 자료에 대한 접근은 권한을 위임 받은 의뢰자측 담당자와 연구자, 그리고 임상시험 관련 담당자만이 가능하다.

13. 통계 분석

13.1표본크기 산정

일차평가지수는 24시간 후 혈중 lactate level이다. 선행연구에서 NMB(Neuromuscular blockage) 의 지속적 투여군과 그렇지 않은 군간의 lactate level은 각각 4.3±3.8, 1.6±1.0 mmol/L 이다 [19]. 두 군간의 차이를 보수적인 관점에서 2.0±3.15로 가정할 경우 alpha=0.05, power=80%의 기준에 필요한 피험자 수는 각 군당 대략 40명이 나타남.

13.2자료분석

**13.2.1 Lactate level**

Primary outcome (Lactate level at 24hr) 같은 경우 정규분포 유무에 따라 two sample t-test 혹은 Wilcoxon rank sum test를 시행한다. 또한 각각의 time point에 따른 (0, 12hr, 24hr) lactate level 분석 방법은 시간에 따른 공변량 분석이 필요한 관계로 mixed model 방법에 의한 분석을 수행한다. 모형의 값들을 구한 후 각각의 모형값에 나오는 AIC (Akaike Information Criteria) 값을 근거로 적합한 모형을 선택할 것이며, 모든 모형의 값들 (=추정된 값들)은 REML (REstricted Maximum Likelihood estimation) 방법을 이용한다.

**13.2.2 Secondary endpoint (mortality, neurological outcome, ICU length of stay, changes of PaO2:FiO2 ratio)**

Mortality, neurologic outcome은 categorical variable이므로 χ2 혹은 Fisher exact tests로 분석한다. ICU length of stay는 연속변수이므로 Mann–Whitney U test 혹은 independent t-test로 분석한다. PaO2:FiO2 ratio의 변화는 linear mixed model 방법에 의한 분석을 이용한다.

**13.2.3 결측치**

Primary outcome(lactate level at 24hr)에 결측치가 발생할 경우해당군에 해당되는 값의 평균 혹은 중위수로 대치한다.

14. 규제 책임

14.1연구자의 책임

연구자는 서명한 모든 합의서와 계획서에 따라 임상시험이 수행되도록 할 책임이 있다. 또한 각 연구자는 연구자 합의서를 이해하고 서명해야 한다.

- 연구자 동의서에 서명하고 이에 따른다.
- 의뢰자가 계획한대로 연구자 회의와 교육 세션에 참여한다.
- 임상시험 계획서에서 기술한 대로 치료 절차를 기꺼이 수행하고 수행할 능력이 있어야 한다.
- 임상시험 계획서의 모든 필수요건을 준수하고 분석에 적합한 자료를 제공한다.
- 모든 연구 특정 절차가 수행되기 전에 각 임상시험 대상자에게서 서면 동의서를 받는다.
- 임상시험 계획상 필요하다면, 병원의 절차를 (임상시험 대상자의 안전과 복리가 훼손되지 않는 한) 조정 한다.
- 중앙 분석 지침을 준수한다.

**15. 기록**

각 연구자는 임상시험 수행과 관련하여 다음의 정확하고, 완벽하며, 가장 최근의 기록을 유지해야 한다. 이러한 기록의 일부 자료는 DCC에서 컴퓨터화된 형식으로도 가능하나 기록을 유지하는 최종 책임은 연구자에게 있다.

**16. 보고**

다음은 연구자의 책임인 보고에 대한 내용이다. 이 표는 보고가 누구에게 보내져야 하고 어느 정도 간격 또는 언제까지 보고해야 하는지를 나타낸다. 이러한 보고서의 일부는 DCC의 도움으로 개발된다. 하지만 보고의 최종 책임은 연구자에게 있다.

**[연구자에게 요구되는 보고]**

| **보고의 종류** | **보고받는 주체** | **보고 기한** |
| --- | --- | --- |
| 중대한 이상반응 | IRB | 해당 지역 규정에 따라 |
|  | DCC | 사망/생명 위협의 경우 근무일수 기준7일 이내 |
|  |  | 그 외 근무일수 기준15일 이내 |
| 중간 보고서 | IRB | 해당 지역 규정에 따라 |
|  |  |  |
|  |  |  |
| 임상시험 계획에 대한 위반 | IRB | 해당 지역 규정에 따라 |
|  | DCC | 근무일수 7일 이내 |
| 최종 요약 보고 | DCC | 1달 이내 |
|  |  |  |

**17. 기록 보존**

필수 문서는 임상시험이 공식적으로 종료되고 최종 결과보고서(모든 종류의 논문 또는 발표물을 포함하며 이에 대한 형식 제한은 없음)의 발행 이후 최소한 3년 동안 보관된다. 기록 보존에 대한 상세한 부분은 지역의 규정에 따른다.

**18. 참고 문헌**

1. McNally B, Robb R, Mehta M, Vellano K, Valderrama AL, Yoon PW, Sasson C, Crouch A, Perez AB, Merritt R, Kellermann A, Centers for Disease C, Prevention. Out-of-hospital cardiac arrest surveillance --- cardiac arrest registry to enhance survival (cares), united states, october 1, 2005--december 31, 2010. Morbidity and mortality weekly report.Surveillance summaries. 2011;60:1-19

2. Lloyd-Jones D, Adams RJ, Brown TM, Carnethon M, Dai S, De Simone G, Ferguson TB, Ford E, Furie K, Gillespie C, Go A, Greenlund K, Haase N, Hailpern S, Ho PM, Howard V, Kissela B, Kittner S, Lackland D, Lisabeth L, Marelli A, McDermott MM, Meigs J, Mozaffarian D, Mussolino M, Nichol G, Roger VL, Rosamond W, Sacco R, Sorlie P, Thom T, Wasserthiel-Smoller S, Wong ND, Wylie-Rosett J. Heart disease and stroke statistics--2010 update: A report from the american heart association. Circulation. 2010;121:e46-e215

3. 질병관리본부 국가심장정지 등록조사사업

4. Group HaCAS. Mild therapeutic hypothermia to improve the neurologic outcome after cardiac arrest.The New England journal of medicine. 2002;346:549-556

5. Bernard SA, Gray TW, Buist MD, Jones BM, Silvester W, Gutteridge G, Smith K. Treatment of comatose survivors of out-of-hospital cardiac arrest with induced hypothermia. The New England journal of medicine. 2002;346:557-563

6. Gooch JL, Suchyta MR, Balbierz JM, Petajan JH, Clemmer TP. Prolonged paralysis after treatment with neuromuscular junction blocking agents. Critical care medicine. 1991;19:1125-1131

7. Segredo V, Caldwell JE, Matthay MA, Sharma ML, Gruenke LD, Miller RD. Persistent paralysis in critically ill patients after long-term administration of vecuronium. The New England journal of medicine. 1992;327:524-528

8. Nielsen N, Wetterslev J, Cronberg T, Erlinge D, Gasche Y, Hassager C, Horn J, Hovdenes J, Kjaergaard J, Kuiper M, Pellis T, Stammet P, Wanscher M, Wise MP, Aneman A, Al-Subaie N, Boesgaard S, Bro-Jeppesen J, Brunetti I, Bugge JF, Hingston CD, Juffermans NP, Koopmans M, Kober L, Langorgen J, Lilja G, Moller JE, Rundgren M, Rylander C, Smid O, Werer C, Winkel P, Friberg H. Targeted temperature management at 33 degrees c versus 36 degrees c after cardiac arrest. The New England journal of medicine. 2013

9. Papazian L, Forel JM, Gacouin A, Penot-Ragon C, Perrin G, Loundou A, Jaber S, Arnal JM, Perez D, Seghboyan JM, Constantin JM, Courant P, Lefrant JY, Guerin C, Prat G, Morange S, Roch A. Neuromuscular blockers in early acute respiratory distress syndrome. The New England journal of medicine. 2010;363:1107-1116

10. Alhazzani W, Alshahrani M, Jaeschke R, Forel JM, Papazian L, Sevransky J, Meade MO.Neuromuscular blocking agents in acute respiratory distress syndrome: A systematic review and meta-analysis of randomized controlled trials. Crit Care. 2013;17:R43

11. Steingrub JS, Lagu T, Rothberg MB, Nathanson BH, Raghunathan K, Lindenauer PK. Treatment with neuromuscular blocking agents and the risk of in-hospital mortality among mechanically ventilated patients with severe sepsis. Critical care medicine. 2013

12. Manthous CA, Hall JB, Kushner R, Schmidt GA, Russo G, Wood LD. The effect of mechanical ventilation on oxygen consumption in critically ill patients.American journal of respiratory and critical care medicine. 1995;151:210-214

13. Gainnier M, Roch A, Forel JM, Thirion X, Arnal JM, Donati S, Papazian L. Effect of neuromuscular blocking agents on gas exchange in patients presenting with acute respiratory distress syndrome. Critical care medicine. 2004;32:113-119

14. Forel JM, Roch A, Marin V, Michelet P, Demory D, Blache JL, Perrin G, Gainnier M, Bongrand P, Papazian L. Neuromuscular blocking agents decrease inflammatory response in patients presenting with acute respiratory distress syndrome. Critical care medicine. 2006;34:2749-2757

15. Peberdy; MA, Ornato; JP, Helm; SL, Thacker; LR, Callaway; CW, Rittenberger; JC, Cocchi; MN, Gaieski; DF, Abella; BS, Donnino; MW, Investigators ftNPARC. Abstract 239: Initial cytokine levels are associated with outcome after cardiac arrest. Circulation. 2013:128:A239

16. Cocchi MN, Miller J, Hunziker S, Carney E, Salciccioli J, Farris S, Joyce N, Zimetbaum P, Howell MD, Donnino MW. The association of lactate and vasopressor need for mortality prediction in survivors of cardiac arrest.Minervaanestesiologica. 2011;77:1063-1071

17. Donnino MW, Miller J, Goyal N, Loomba M, Sankey SS, Dolcourt B, Sherwin R, Otero R, Wira C. Effective lactate clearance is associated with improved outcome in post-cardiac arrest patients. Resuscitation. 2007;75:229-234

18. Salciccioli JD, Cocchi MN, Rittenberger JC, Peberdy MA, Ornato JP, Abella BS, Gaieski DF, Clore J, Gautam S, Giberson T, Callaway CW, Donnino MW. Continuous neuromuscular blockade is associated with decreased mortality in post-cardiac arrest patients. Resuscitation. 2013;84:1728-1733

19. De Jonghe B, Sharshar T, Lefaucheur JP, Authier FJ, Durand-Zaleski I, Boussarsar M, Cerf C, Renaud E, Mesrati F, Carlet J, Raphael JC, Outin H, Bastuji-Garin S, Groupe de Reflexion et d'Etude des Neuromyopathies en R. Paresis acquired in the intensive care unit: A prospective multicenter study. JAMA : the journal of the American Medical Association. 2002;288:2859-2867
